# Pseudo-capacitive and kinetic enhancement of metal oxides and pillared graphite composite for stabilizing battery anodes

**DOI:** 10.1038/s41598-022-15789-0

**Published:** 2022-07-15

**Authors:** Yongguang Luo, Lingling Wang, Qian Li, Jungsue Choi, G. Hwan Park, Zhiyong Zheng, Yang Liu, Hongdan Wang, Hyoyoung Lee

**Affiliations:** 1 Center for Integrated Nanostructure Physics (CINAP), Institute for Basic Science (IBS), 2066 Seoburo, Jangan-gu, Suwon, 16419 Republic of Korea; 2 Department of Chemistry, Sungkyunkwan University, 2066 Seoburo, Jangan-gu, Suwon, 16419 Republic of Korea; 3 Department of Applied Environmental Science, College of Engineering, Kyunghee University, Yongin, 17104 Republic of Korea; 4 Department of Biophysics, Sungkyunkwan University, 2066 Seoburo, Jangan-gu, Suwon, 16419 Republic of Korea; 5 Creative Research Institute, Sungkyunkwan University, 2066 Seoburo, Jangan-gu, Suwon, 16419 Republic of Korea; 6 BYD Company Ltd., 1301 Shenshan Road, Pingshan District, Shenzhen, 518122 China

**Keywords:** Batteries, Chemical engineering

## Abstract

Nanostructured TiO_2_ and SnO_2_ possess reciprocal energy storage properties, but challenges remain in fully exploiting their complementary merits. Here, this study reports a strategy of chemically suturing metal oxides in a cushioning graphite network (SnO_2_[O]rTiO_2_-PGN) in order to construct an advanced and reliable energy storage material with a unique configuration for energy storage processes. The suggested SnO_2_[O]rTiO_2_-PGN configuration provides sturdy interconnections between phases and chemically wraps the SnO_2_ nanoparticles around disordered TiO_2_ (SnO_2_[O]rTiO_2_) into a cushioning plier-linked graphite network (PGN) system with nanometer interlayer distance (~ 1.2 nm). Subsequently, the SnO_2_[O]rTiO_2_-PGN reveals superior lithium-ion storage performance compared to all 16 of the control group samples and commercial graphite anode (keeps around 600 mAh g^−1^ at 100 mA g^−1^ after 250 cycles). This work clarifies the enhanced pseudo-capacitive contribution and the major diffusion-controlled energy storage kinetics. The validity of preventing volume expansion is demonstrated through the visualized image evidence of electrode integrity.

## Introduction

Multiple crises, including environmental pollution, depletion of primary energy sources, and inadequate storage of clean energy, are currently hindering societal development^1,2^. The modern lithium-ion battery (LIB) presents a potential solution for these vital concerns by reforming traditional energy systems^3,4^. However, the present LIB configuration is unable to support recent technological advancements in electric vehicles, smart devices, and emerging technologies^5^. This study aims to explore a more advanced design, specifically by replacement of the traditional graphite anode, in order to obtain materials with higher energy storage ability.

The common metal oxides, tin dioxide (SnO_2_) and titanium dioxide (TiO_2_), have been recognized as encouraging and promising LIB anode candidates due to their various advantages^6–8^. Initially, SnO_2_ drew immense attention mainly due to its higher theoretical lithium-ion (Li^+^) storage capacity (1494 mAh g^−1^, Li_4.4_Sn) relative to graphite (372 mAh g^−1^, LiC_6_), while being inexpensive and naturally abundant^9^. Furthermore, the electrochemical potential for reversible Li^+^ storage in SnO_2_ is around 0.6 V vs. Li/Li^+^, which is a preferred characteristic in an anode. However, practical utilization of SnO_2_ as a LIB anode is still far off due to the severe capacity decay upon cycling induced by considerable volume change (up to 350%) during charge/discharge processes^10^. On the other hand, TiO_2_ attracts extensive energy storage research interest because of its several virtues, which are superior to graphite. When TiO_2_ is applied as a LIB anode, it offers an extremely low volume change during cycling (< 4%, rather than the 10% of the graphite case), less solid-electrolyte-interface (SEI) formation, and is non-toxic and inexpensive^11^. Nonetheless, its low theoretical storage capacity (168 mAh g^−1^ in Li_0.5_TiO_2_ form) is the fatal aspect that impedes further development of TiO_2_ as a substitute for commercialized graphite anodes.

Over the past few years, two main approaches for improving the energy storage performance of TiO_2_-based materials have been suggested: the self-doping of electro-conducting Ti^3+^ in TiO_2_ nanostructures and the compositing of TiO_2_ with carbon^12–15^. These methods result in better electrical conductivity, enhanced Li^+^ transportation kinetics, and improved pseudocapacitive contribution of TiO_2_-based LIB anodes. Nevertheless, the upgraded designation of electrode configuration still only reached around 200–300 mAh g^−1^ due to the intrinsic storage capacity limit of TiO_2_. Therefore, research communities have attempted to design TiO_2_ and SnO_2_ composites to compensate for their respective limitations and boost energy storage performance^16–20^. Mullins et al. first reported the improved cyclability and higher coulombic efficiency of a TiO_2_-supported SnO_2_ nanocomposite as opposed to pure SnO_2_ nanoparticles as an anode in LIB, achieving about 320 mAh g^−1^ storage performance^16^. Zheng et al. tried Sn-doping strategies in mesoporous TiO_2_ film to perform efficient ion transport and maintain electrode structural stability^17^. Meanwhile, these preliminary endeavors still suffered from capacity decay, which originated from the volume change of SnO_2_ during cycling and only attained limited improvement in energy storage performance. Consequently, researchers have started to fabricate TiO_2_–SnO_2_ composites in a one-dimensional nanotube or layered sandwich structure to release the mechanical strain from SnO_2_ and preserve the structural integrity of the electrode. The anodically constructed TiO_2_–SnO_2_ nanotube composite by Madian et al. possesses about 400 mAh g^−1^ Li^+^ storage capacity^18^. Moreover, Choi suggested that the microcone-morphology Ti and Sn oxides complex assembled by growing SnO_2_ species between TiO_2_ microcone layers also exhibited enhanced energy storage properties^20^. These further efforts lifted the reversible Li^+^ storage ability of Ti/Sn-based oxide material to around 400 mAh g^−1^ through the elaborate design of material configuration.

Queries may arise where the route will further boost the energy storage capability of Ti/Sn-based oxide anodes. Nano-engineering structural design and incorporation of Sn species into the Ti/Sn-based oxides have contributed to alleviating SnO_2_ volume expansion. To further strengthen the TiO_2_–SnO_2_ materials, we suggest a different strategy of introducing the unique and efficient carbon-based cushioning material system and chemically assembling the cushioning material system by including covalently bonded TiO_2_ and SnO_2_. Through creating the sufficient widely preserved two-dimensional (2D) nano-space and firm ether covalent bonding (–[O]–) interconnections among each species, the proposed structure is highly promising to thoroughly release the volume change strain and buffer the severe volume expansion of SnO_2_ during the lithiation process. It sustains long-term electrode integrity with the high energy storage performance of Ti/Sn oxides-based LIB anodes. Moreover, the electrochemically active plier-like linker molecules in the carbon-based cushioning system provide enriched Li^+^ storage sites. The nano-sized zero-dimensional TiO_2_ and SnO_2_ particles and cushioning material of the graphite network expose a large area of active surface, further facilitating the pseudocapacitive contribution in enhancing energy storage performance.

Herein, we present a new SnO_2_[O]rTiO_2_ chemically wrapped with a graphite network (PGN) for preventing metal oxides battery anodes’ volume expansion. The covalent-bonded SnO_2_ on the reduced TiO_2_ (SnO_2_[O]rTiO_2_) is designed as the first shield to prevent severe volume expansion and pulverization of SnO_2_ during the energy storage process. The PGN is introduced to chemically anchor SnO_2_[O]rTiO_2_ while acting as a buffer membrane to cushion and prevent the pulverization of SnO_2_[O]rTiO_2_. In order to realize chemical bonding in SnO_2_[O]rTiO_2,_ we produce a hydroxyl-rich (-OH) surface on TiO_2_ by breaking the Ti-oxygen bond, resulting in a reduced TiO_2_ (rTiO_2_)^8,21–25^. To effectively tackle volume change and pulverization concerns, we tried to adjust and achieve the proper ratio of SnO_2_ (~ 3–4 nm) and TiO_2_ (~ 5 nm). Conjugated organic linker molecules are used to suture SnO_2_[O]rTiO_2_ into the PGN cushioning membrane while simultaneously supplying extra energy storage sites. The evidence of volume expansion mitigation was also investigated to demonstrate the validity of the proposed configuration's cushioning ability. The concept of building up the chemical interconnections and the cushioning PGN system with electrochemically active properties provides a new strategy for boosting the energy storage of metal oxides or large volume variation electrode materials.

## Experimental section

### Construction of SnO_2_[O]rTiO_2_-PGN and the control group samples

**Figure 1:**
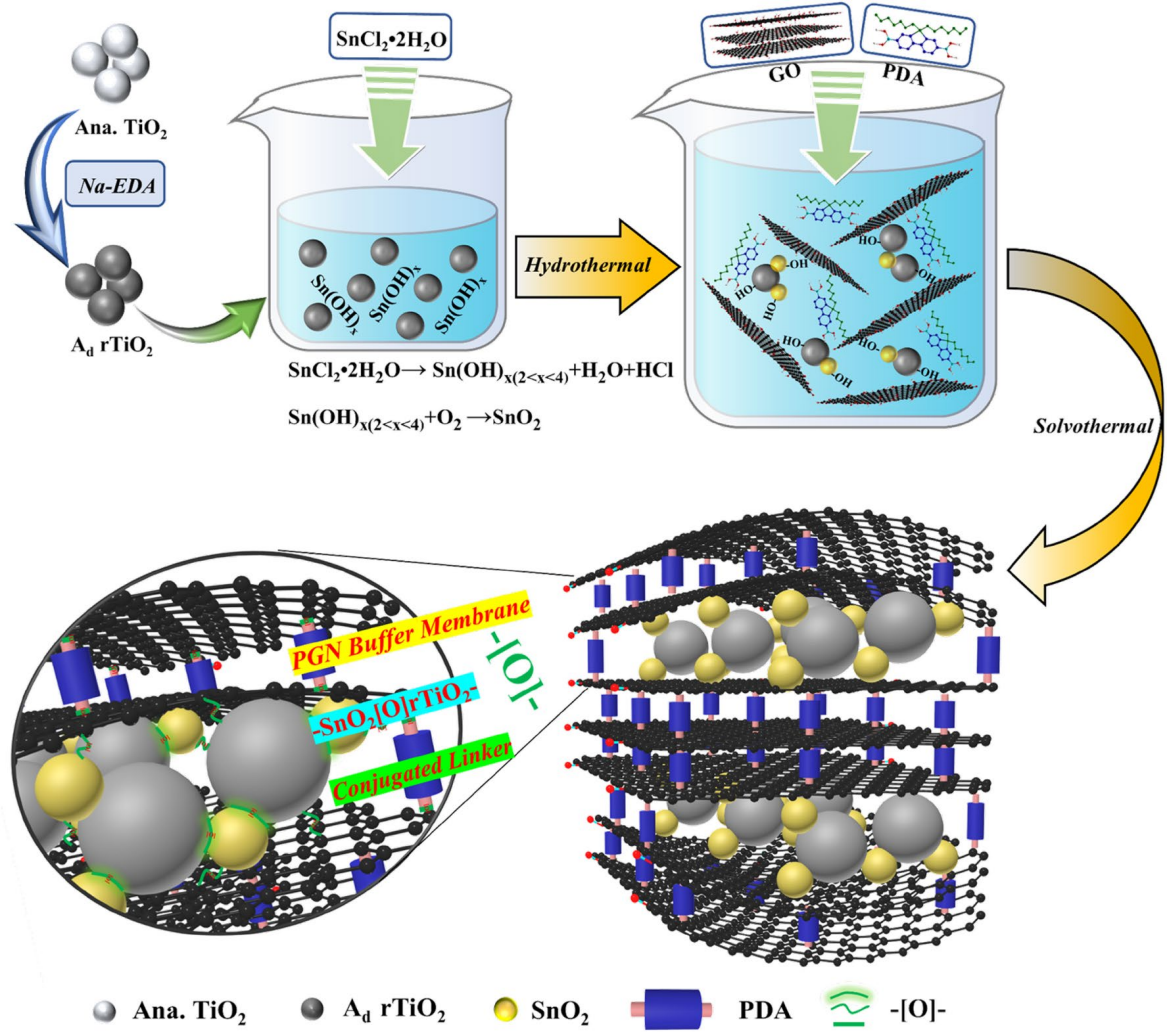
Synthesis route illustrations of SnO_2_[O]rTiO_2_-PGN construction.

**Figure 2:**
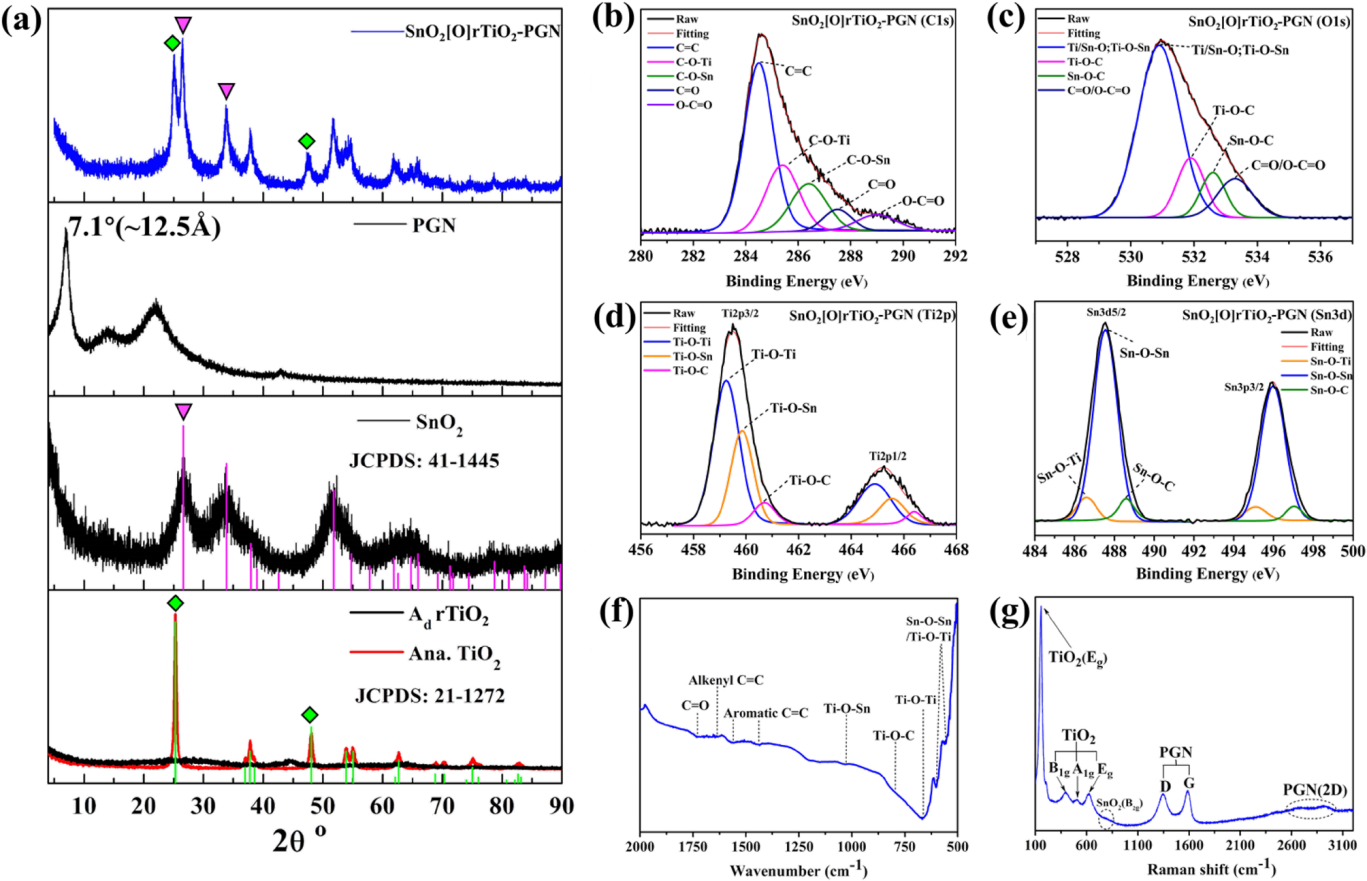
Structure and bonding state characterization of SnO_2_[O]rTiO_2_-PGN: (**a**) XRD patterns of SnO_2_[O]rTiO_2_-PGN, PGN, SnO_2,_ and A_d_, Ana. TiO_2_. (**b**–**e**) SnO_2_[O]rTiO_2_-PGN XPS spectra of C1s, O1s, Ti2p, and Sn3d, respectively. The deconvoluted bonding species are labeled. (**f**) FT-IR and (**g**) Raman patterns of SnO_2_[O]rTiO_2_-PGN.

To fabricate the proposed configuration of SnO_2_[O]rTiO_2_-PGN, we design the process depicted in Fig. 1 and perform the synthesis stepwise. The synthesis approach consists of three procedures, which include A_d_ rTiO_2_ preparation, SnO_2_ growth on A_d_ rTiO_2,_ and the introduction of cushioning PGN. First, we enrich the A_d_ rTiO_2_ with hydroxyl groups (–OH) through sodium metal ethylenediamine (Na-EDA) solution treatment to pristine anatase (Ana.) TiO_2_ and concurrently transform the intact crystalline structure to a Ti^3+^ self-doped amorphous state^21^. The X-ray photoelectron spectroscopy (XPS) of A_d_ and Ana. TiO_2_ reveals the enriched –OH and self-doping of Ti^3+^ species in A_d_ rTiO_2_, as shown in Supplementary Fig. S1 and Supplementary Table S1 of Supporting Information^22^. Additionally, the phase transformation of Ana. to A_d_ rTiO_2_ is apparent in the X-ray powder diffraction (XRD) pattern at the bottom of Fig. 2a, which shows the complete disappearance of crystalline peaks in A_d_ rTiO_2_. The amorphous state indicates the deeply reduced status of A_d_ rTiO_2,_ which can maximize the proportion of Ti–OH and Ti^3+^ species and subsequently facilitate the important chemical bonding between SnO_2_ and A_d_ TiO_2_. Afterward, we deposit SnO_2_ nanoparticles on A_d_ rTiO_2_ through hydrolysis of stannous chloride dihydrate (SnCl_2_·2H_2_O) and condensation reactions among –OH groups under hydrothermal conditions^26^. During this process, we hold the SnO_2_ nanoparticles on A_d_ rTiO_2_ by forming a strong –[O]– covalent bond (Ti–O–Sn), as evidenced by the deconvolution peaks of XPS Ti2p and Sn3d spectra in Fig. 2d,e. The XRD spectrum of the synthesized SnO_2_ confirms its rutile phase structure, matching with standard JCPDS data (41-1445), as shown in Fig. 2a. Moreover, the XPS spectra of the synthesized SnO_2_ control sample demonstrate the presence of Sn–OH and Sn^2+^ species (Supplementary Fig. S2a–c). Subsequently, we incorporate the assembled SnO_2_[O]rTiO_2_ into a cushioning PGN membrane system with the chemical bonding linkages to each species after conducting solvothermal reactions^27^. The PGN buffering membrane is generated after the condensation reactions among B–OH at PDA molecules and C–OH at oxidized graphene layers under the solvothermal reaction circumstance. The PDA pillar molecules are covalently bonded with graphite interlayers through B–O–C bonding that can provide the cushioning pre-volume. The eventual structure of this SnO_2_[O]rTiO_2_-PGN composite is presented in Fig. 1.

First, 100 ml ethylenediamine anhydrous (TCI, > 98%) was injected into a 300 ml three-neck round-bottom-flask (RBF) under an N_2_ atmosphere, followed by adding 2.3 g sodium metal (Alfa Aesar, > 99.8%) while gently stirring for thirty minutes. An ice bath was installed below the RBF to absorb the released heat during stirring. After that, 1 g anatase TiO_2_ (Sigma-Aldrich) was weighed and added to the RBF. The reduction process was carried out at room temperature and in an N_2_-flowing atmosphere for five days to achieve sufficient Ti^3+^ self-doping and Ti–OH generation. A 35% HCl (Matsunoen Chemicals LTD.) solution was added dropwise and slowly until a neutral suspension was obtained for quenching the reduction reaction. The reduced A_d_ rTiO_2_ product was washed with deionized water several times, followed by centrifugation, filtration, and vacuum drying. Subsequently, 100 mg A_d_ rTiO_2_ powder was dispersed in 30 ml water by one-hour tip sonication with stirring. Then, 0.86 g (~ 3.8 mmol) stannous chloride dihydrate (Sigma-Aldrich, ACS reagent, 98%) and 0.5 ml 35% HCl were added to the A_d_ rTiO_2_ dispersion, followed by 30 min of stirring. Next, to construct the SnO_2_[O]rTiO_2_ composite, the suspension was added into a Teflon container, sealed in the autoclave, and a hydrothermal reaction was conducted at 160 °C for two hours to obtain the SnO_2_[O]rTiO_2_ composite. The SnO_2_[O]rTiO_2_ product was washed with water, filtrated, dried under a vacuum, and redispersed into 20 ml methanol. The GO was prepared from graphite powder (Sigma-Aldrich, < 20 μm, synthetic) by modifying Hummer’s method^28^. The 10 ml beforehand GO dispersion (5 mg ml^−1^) and 0.5 mmol 9,9-Dioctylfluorene-2,7-diboronic acid (PDA) were added into the SnO_2_[O]rTiO_2_ dispersion followed by 30 min homogenization. The mixture underwent a solvothermal reaction at 135 °C for 12 h in order to build the SnO_2_[O]rTiO_2_-PGN configuration. Afterward, the synthesized SnO_2_[O]rTiO_2_-PGN composite was washed with methanol along with filtration and vacuum drying.

Control group sample Number 4, SnO_2_, was synthesized by adding 0.86 g (~ 3.8 mmol) stannous chloride dihydrate and 0.5 ml 35% HCl into 30 ml water. The mixture was stirred for 30 min, followed by 2 h of hydrothermal reaction at 160 °C. The SnO_2_ nanoparticle powder product was acquired after washing with water, centrifuging, filtrating, and vacuum drying. Control group sample Number 5, PGN, was synthesized by adding 10 ml beforehand GO dispersion and 0.5 mmol PDA into 20 ml methanol. A solvothermal reaction was conducted at 135 °C for 12 h. Control group samples Numbers 6 and 7, Ana-PGN and A_d_-PGN, were synthesized starting from anatase and A_d_ rTiO_2,_ respectively. The anatase or A_d_ rTiO_2_ powder was mixed with 10 ml beforehand GO dispersion, 0.5 mmol PDA, and 20 ml methanol along with 30 min stirring, then the solvothermal reaction was carried out at 135 °C for 12 h. Control group sample Number 8, SnO_2_-PGN, followed the SnO_2_[O]rTiO_2_-PGN synthesis approach but omitted the addition of A_d_ rTiO_2_. Control group samples Number 9 and 10, Ana-SnO_2_ and A_d_-SnO_2,_ followed the SnO_2_[O]rTiO_2_ synthesis approach but started from anatase and A_d_ rTiO_2_, respectively. The A_d_-SnO_2_ was the same as SnO_2_[O]rTiO_2_ construction. Control group sample Number 11, A_d_/SnO_2_, was prepared by dispersing the synthesized SnO_2_ with A_d_ rTiO_2_, stirring for 30 min, then filtrating to get the physically mixed A_d_/SnO_2_. Control group sample Number 13, A_d_/SnO_2_-PGN, was obtained by adding 10 ml beforehand GO dispersion and 0.5 mmol PDA into 30 ml A_d_/SnO_2_ methanol dispersion and conducting a solvothermal reaction at 135 °C for 12 h. Control group samples Numbers 12 and 14, Ana-SnO_2_-PGN and A_d_-SnO_2_-G, were prepared using the synthesizing method of SnO_2_[O]rTiO_2_-PGN but replacing the A_d_ rTiO_2_ with anatase TiO_2_ and removing the addition of PDA, respectively. Control group samples Numbers 15, 16, and 17, SnO_2_[O]rTiO_2_-PGN(II), SnO_2_[O]rTiO_2_-PGN(III), and SnO_2_[O]rTiO_2_-PGN(IV), were synthesized by following the same procedure of SnO_2_[O]rTiO_2_-PGN but adjusting the mass ratio of A_d_: SnO_2_.

### Material characterization

The XRD data were measured by Rigaku Smart Lab JD3643N diffractometer with Cu Kα radiation (λ = 1.5406 Å). The investigated samples' elemental compositions and valence states were checked through XPS (ESCA 2000, VG Microtech) and Raman spectroscopy (Renishaw 2000 system). Additionally, FTIR spectra were collected by a Bruker Vertex 70/80 FTIR spectrometer. ICP-OES measurement was conducted by Agilent Technologies 5100 ICP-OES with 189.9 nm wavelength for Sn and 336.1 nm for Ti element. The materials’ morphologies and microstructures were acquired by SEM (JSM 7000F, JEOL) and TEM (JEOL JEM-2100F). BET measurements were determined using nitrogen sorption at a liquid-nitrogen temperature and a BELSORP-max (MP) instrument.

### Electrochemical measurements

To prepare the LIB anode electrode, active materials, polyvinylidene difluoride (PVDF powder, Sigma-Aldrich, average M_w_ ~ 534,000 by GPC), vapor-grown carbon fibers (VGCF™-H, Showa Denko K. K.) conductivity agent were mixed at an 8:1:1 mass ratio in mortar. An appropriate amount of 1-methyl-2-pyrrolidinone (NMP, Sigma-Aldrich) was added to the mixture as a solvent to generate a uniform slurry. The slurry was cast on copper foil (18 µm thickness) by a doctor blade, and the electrodes were placed in a vacuum oven for 24 h under 60 °C to remove residual NMP. The electrodes were cut into a circle 12 or 8 mm in diameter and fabricated into a CR2032 coin-type half cell in a specialized dry room (dew point maintained at around – 50 °C, moisture level less than 100 ppm). All electrodes have been coated with nearly the same coating thickness, and the mass loading of the electrodes was around 1–2 mg/cm^2^. The counter/reference electrode was made up of a pure lithium metal foil that was cut into a 15 mm circle. Celgard 2400 polypropylene was used as the separator. Ethylene carbonate/diethyl carbonate 1:1 ratio (v/v) containing 1 M LiPF_6_ served as the electrolyte. Around 0.2 ml electrolyte was added to one cell. Specific capacity was calculated from the mass of the active material in the electrodes. The CV data were measured using a VMP3 electrochemical workstation (Bio-Logic Science). GCD and rate-capability data were collected at room temperature using a WonA Tech WBCS3000 Automatic Battery Cycler at various current conditions and over a potential range of 0–3 V.

## Results and discussion

### SnO_2_[O]rTiO_2_-PGN synthesis and structure confirmation

The effectiveness of the configuration in energy storage is attributed to the contribution of PGN buffer membrane, unique –SnO_2_[O]rTiO_2_– structure, and potentially electrochemical active conjugated linkers. The PGN structure keeps obviously expanded interlayer spacing (~ 12.5 Å, as shown in Fig. 2a, around fourfold those of graphite or other types of π–π stacked carbon) in comparison with conventional reduced graphene oxide layers and provides a promising ability to release volume expansion strains by the existence of pre-volume and pillars between cross-linked graphene sheets. The interlayer d spacing of PGN is derived from the measured XRD data, as shown and labeled in Fig. 2a according to Bragg’s law^29^. We also collect the XPS data of PGN and show the deconvoluted peaks' chemical environments in Supplementary Fig. S2d–f for the confirmation of PGN chemical bonding environments.

To verify the proposed structure and the expected improvement of the properties of SnO_2_[O]rTiO_2_-PGN, we synthesize 17 samples, including SnO_2_[O]rTiO_2_-PGN and 16 control samples, and carry out several characterization measures. The list of prepared samples and the corresponding description of each sample are shown in Table 1. The XRD pattern of SnO_2_[O]rTiO_2_-PGN is shown in the uppermost plot of Fig. 2a, which presents the three components in the composite, the bump before 10° of PGN, rTiO_2_ peaks (green rhombuses), and SnO_2_ peaks (magenta inverted triangles). Furthermore, the XRD spectra of other control samples are given in Supplementary Fig. S3. The XPS chemical, environmental analyses of SnO_2_[O]rTiO_2_-PGN on C1s, O1s, Ti2p, and Sn3d are presented in Fig. 2b–e, and the full XPS spectrum of SnO_2_[O]rTiO_2_-PGN is shown in Supplementary Fig. S4d. The deconvoluted species of SnO_2_[O]rTiO_2_-PGN C1s indicate the presence of C–O–Ti and C–O–Sn, which further confirms that rTiO_2_ and SnO_2_ are chemically anchored on the PGN, as shown in Fig. 2b. The peak deconvolution refers to the NIST databases and literature^30–32^. Besides, the XPS positions of C–O–Ti and C–O–Sn are consistent with the concept of the Pauling scale electronegativity (Supplementary Table S2) trend, in that the higher electronegativity of Sn (1.96) more effectively decreases the C electron cloud density than Ti (1.54), weakens the shielding effect around C and ultimately improves the binding energy of C–O–Sn relative to that of C–O–Ti in C1s spectra^33,34^.

**Table 1:** The list of investigated samples in this study and the corresponding description of each sample.

Numb	Sample name	Description (composition of recipe)	Reversible Cap. (at 100th cycle, mAh/g)	Notes
1	SnO_2_[O]rTiO_2_-PGN	Optimized design (A_d_: SnO_2_ = 50:25, in mass of mg)	505	Target design
2	Ana. TiO_2_	Pristine TiO_2_ of anatase crystalline phase	17	Control Group A (Single-phase)
3	A_d_ rTiO_2_	Disordering rTiO_2_ of reduced anatase crystalline phase	18	
4	SnO_2_	Hydrothermal synthesized SnO_2_	71	
5	PGN	Pillared graphite network	161	
6	Ana-PGN	Composite of anatase TiO_2_ and PGN	115	Control Group B (Binary-phases)
7	A_d_-PGN	Composite of A_d_ rTiO_2_ and PGN	116	
8	SnO_2_-PGN	Composite of SnO_2_ and PGN	462	
9	Ana-SnO_2_	Composite of anatase TiO_2_ and SnO_2_	49	
10	A_d_-SnO_2_	Composite of A_d_ rTiO_2_ and SnO_2_	112	
11	A_d_/SnO_2_	Mixing of A_d_ rTiO_2_ and SnO_2_	27	
12	Ana-SnO_2_-PGN	Composite of anatase TiO_2_, SnO_2,_ and PGN	216	Control Group C (Ternary-phases)
13	A_d_/SnO_2_-PGN	Composite of mixed A_d_/SnO_2_ and PGN	299	
14	A_d_-SnO_2_-G	Composite of A_d_ rTiO_2_, SnO_2_ and rGO	275	
15	SnO_2_[O]rTiO_2_-PGN(II)	Target design optimization (A_d_: SnO_2_ = 50:50)	461	
16	SnO_2_[O]rTiO_2_-PGN(III)	Target design optimization (A_d_: SnO_2_ = 50:75)	469	
17	SnO_2_[O]rTiO_2_-PGN(IV)	Target design optimization (A_d_: SnO_2_ = 30:50)	489	

Similarly, we identify the Ti–O–C and Sn–O–C in SnO_2_[O]rTiO_2_-PGN O1s, Ti2p, and Sn3d spectra, respectively (Fig. 2c–e)^15,20,35^. In order to trace the changes during the SnO_2_[O]rTiO_2_-PGN construction process, we compare the XPS spectra of various control samples in different synthesis stages and material combinations. The C–O–Ti species region in C1s (285–286 eV) and O1s (near 532 eV) exhibits higher intensity in A_d_-PGN than Ana-PGN, indicating that the hydroxyl-rich A_d_ rTiO_2_ more easily forms covalent bonds with PGN than pristine crystalline Ana. TiO_2_ (Supplementary Fig. S4a–c). Additionally, we observe the binding energy of Sn3d and O1s peak upward, shifting to a higher value in SnO_2_[O]rTiO_2_-PGN and SnO_2_-PGN than SnO_2_, which is attributed to the arising of Sn–O–C (Supplementary Fig. S4d–f). The phenomena also can be explained by the Pauling electronegativity property of Sn (1.96) and C (2.55), which makes the binding energy of Sn–O–C in SnO_2_[O]rTiO_2_-PGN and SnO_2_-PGN higher than Sn–O–Sn in SnO_2_. Similarly, Ti–O–Sn formation can downward shift the Sn3d peak to a lower binding value in SnO_2_[O]rTiO_2_-PGN and A_d_-SnO_2_ than other control group cases that are hard to generate the chemical bonding (Supplementary Fig. S5a–d). Additionally, we apply measurements of Fourier-transform infrared (FT-IR) and Raman spectroscopy to characterize the bonding species and chemical environments of SnO_2_[O]rTiO_2_-PGN. As presented in Fig. 2f, the FT-IR spectrum of SnO_2_[O]rTiO_2_-PGN displays the transmittance peaks of Ti–O–C (795 cm^−1^), Ti–O–Sn (1030 cm^−1^), C=C of PGN (1560 and 1635 cm^−1^), which again evidence the essential chemical interconnections in SnO_2_[O]rTiO_2_-PGN composite. The detailed FT-IR vibration peak assignments are summarized in Table S3 using values from the literature^22,31,36–39^. Moreover, Raman data reveals the E_g_ (151 and 621 cm^−1^), B_1g_ (395 cm^−1^) and A_1g_ (508 cm^−1^) bands of TiO_2_, D (1347 cm^−1^), G (1586 cm^−1^), and 2D (2600–3000 cm^−1^) bands of PGN, as shown in Fig. 2g ^40^. The SnO_2_ Raman signals are hard to distinguish in SnO_2_[O]rTiO_2_-PGN due to the overlapping with TiO_2_ signals, only appearing as a weak bump of SnO_2_ B_2g_ bands near 770 cm^−1^^41^. The Raman spectrum of a single component in SnO_2_[O]rTiO_2_-PGN (TiO_2_, SnO_2,_ and PGN) is shown in Supplementary Fig. S6. The composition of SnO_2_[O]rTiO_2_-PGN is measured by inductively coupled plasma-optical emission spectrometry (ICP-OES) and given in Supplementary Table S4.

**Figure 3:**
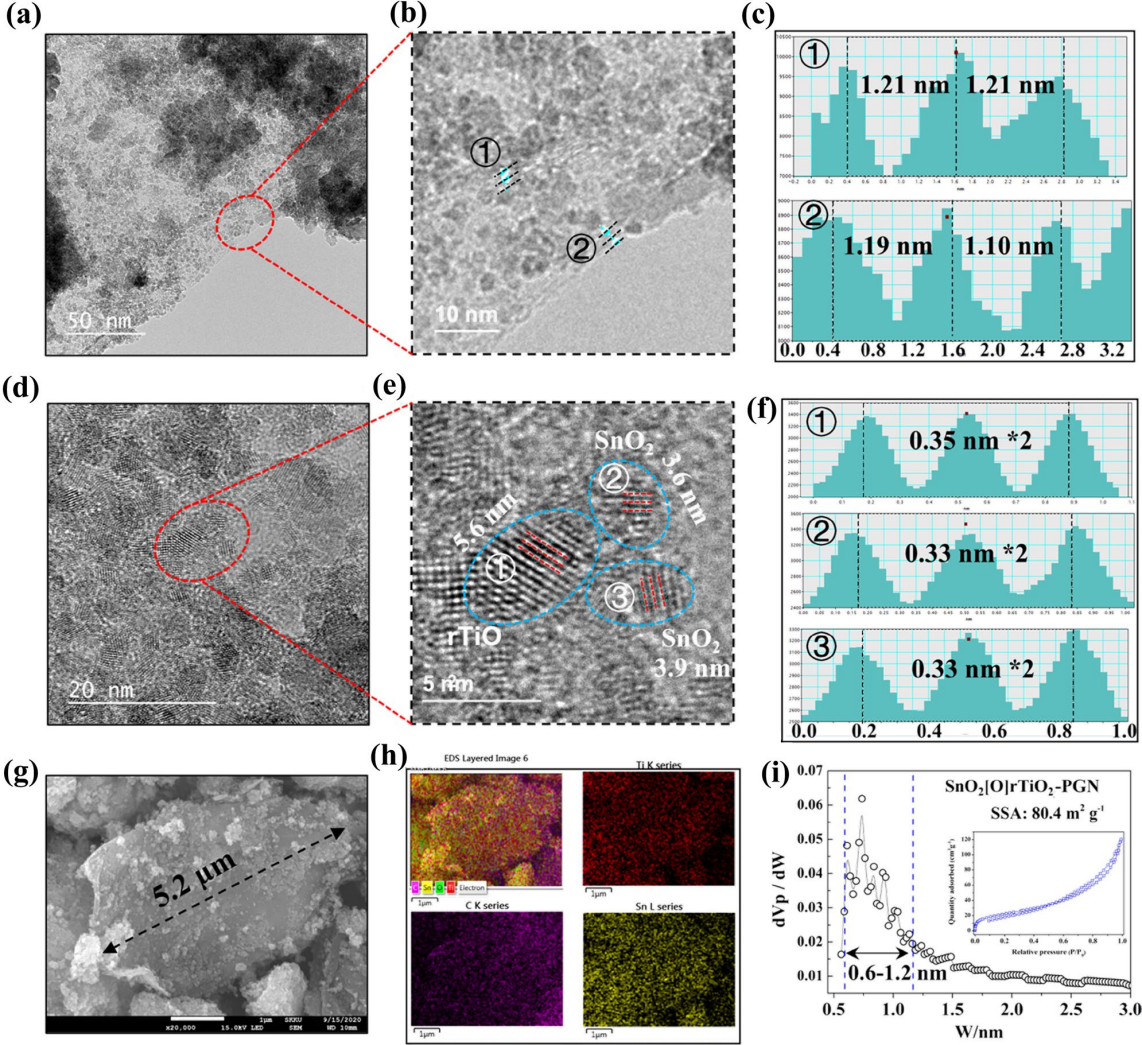
Morphology and microstructures of SnO_2_[O]rTiO_2_-PGN: (**a**) and (**d**) TEM images collected under lower magnification to present the overall structure. The areas in red circles are magnified in (**b**) and (**e**), respectively. The lattice spacing values of the number points are shown in the (**c**) and (**f**) contrast profiles. (**g**) SnO_2_[O]rTiO_2_-PGN SEM image. (**h**) EDS mapping data. (**i**) BET measurement information. Inset, N_2_ adsorption/desorption profiles.

We further characterize the morphology and structure of SnO_2_[O]rTiO_2_-PGN through a transmission electron microscope (TEM), scanning electron microscope (SEM), and Brunauer–Emmett–Teller analyses (BET) to validate the suggested configuration. As shown in Fig. 3a, the large-area 2D PGN sheets effectively hold the SnO_2_[O]rTiO_2_ nanocomposites. The red region of Fig. 3a is enlarged in Fig. 3b. The interlayer d-spacing of PGN is around 1.1–1.2 nm, according to the contrast profiles in Fig. 3c, which are consistent with the XRD data of PGN. Furthermore, the higher-magnification image of SnO_2_[O]rTiO_2_-PGN in Fig. 3d presents the densely loaded SnO_2_[O]rTiO_2_ nanoparticles on PGN layers and indicates the adequate loading amount of active metal oxides material. The red circled region of Fig. 3d is enlarged in Fig. 3e to differentiate between the rTiO_2_ and SnO_2_ nanoparticles. It is noted that the SnO_2_ nanoparticles (3.6–3.9 nm) attach to the surface of rTiO_2_ nanoparticles (5.6 nm), confirming the success of introducing Ti–O–Sn interconnections between SnO_2_ and rTiO_2_.The contrast profiles in Fig. 3f identify the rTiO_2_ and SnO_2_ species according to the lattice fringes difference of 0.33 nm (110) plane for tetragonal rutile SnO_2_ and 0.35 nm (101) plane for tetragonal anatase TiO_2_^21,42^. Based on the SEM measurements, we discover that the SnO_2_[O]rTiO_2_-PGN reveals thin platelet morphology and maintains a long-range lateral size around 5.2 µm (Fig. 3g). The SEM images of every phase in SnO_2_[O]rTiO_2_-PGN are shown in Supplementary Fig. S7. Furthermore, the SEM energy dispersive X-ray (EDX) mapping of SnO_2_[O]rTiO_2_-PGN presents the uniform distribution of Ti and Sn on PGN, which implies the uniformity of rTiO_2_ and SnO_2_ loading (Fig. 3h). The BET analysis of the SnO_2_[O]rTiO_2_-PGN composite shows that the micropore size is dominantly in the range 0.6–1.2 nm, which is consistent with PGN configuration, and the specific surface area (SSA) reaches 80.4 m^2^ g^−1^ (Fig. 3i). The wide 2D microporous structure, broad interlayer distance, and uniform distribution of SnO_2_[O]rTiO_2_ in the PGN cushioning system can effectively support the energy storage electrochemical dynamics and high-quality electrode manufacturing.

### Electrochemical performance investigation

**Figure 4:**
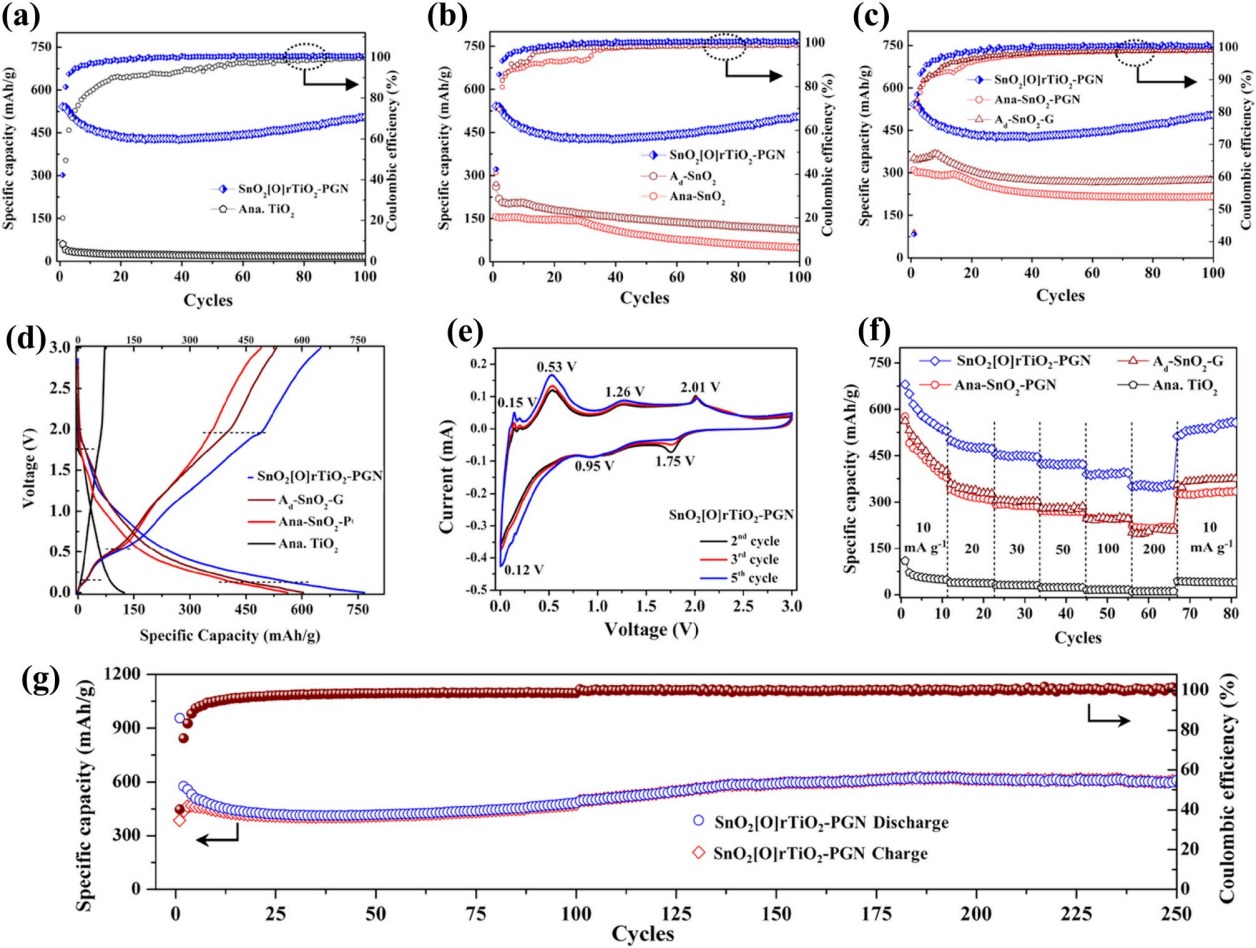
Electrochemical performance investigation. (**a**–**c**) GCD cycling performance comparisons among SnO_2_[O]rTiO_2_-PGN with single/binary/ternary phase samples under 100 mA g^−1^. (**d**) CD profiles of SnO_2_[O]rTiO_2_-PGN, A_d_-SnO_2_-G, Ana-SnO_2_-PGN, and Ana. TiO_2_ at a current density of 10 mA g^−1^. (**e**) CV pattern under different cycles at 0.1 mV s^−1^ reveals the electrochemical processes in the first five cycles. (**f**) Charging capacity profiles under 10–200 mA g^−1^. (**g**) continuing cycling test of the SnO_2_[O]rTiO_2_-PGN LIB anode at a current density of 100 mA g^−1^.

In order to demonstrate the energy storage superiority of SnO_2_[O]rTiO_2_-PGN, we perform galvanostatic charge–discharge (GCD) measurements under 100 mA g^−1^ to all 17 of the synthesized samples (SnO_2_[O]rTiO_2_-PGN and control group) for a comprehensive comparison. We divide the control group into three categories for the convenience of understanding, A (Single-phase), B (Binary-phases), and C (Ternary-phases), as listed in Table 1. As shown in Fig. 4a–c, Supplementary Fig. S8 and Table 1, the reversible cycling capacities of all of the control group samples are inferior to that of the SnO_2_[O]rTiO_2_-PGN in 100 cycles. The SnO_2_[O]rTiO_2_-PGN anode maintains 100% coulombic efficiency (CE) after the 20th cycle, which exhibits the most stable and highest CE% among all the control group samples. In Fig. 4a and Supplementary Fig. S8a, the single-phase samples fail due to their intrinsic limitations, like low conductivity of metal oxides, large volume variation (SnO_2_) and SEI formation, which result in low energy storage ability or capacity fading. The binary-phase products A_d_-PGN and Ana-PGN exhibit higher performance than A_d_ and Ana. rTiO_2_ due to the incorporation with PGN (Fig. 4b and Supplementary Fig. S8b). Furthermore, we can derive the merits of Ti–O–Sn chemical bonding in A_d_-SnO_2_ (equal to “SnO_2_[O]rTiO_2_”) to the energy storage performance enhancements from the GCD profiles of various TiO_2_ and SnO_2_ combinations. The reversible specific capacity increases from physically mixed A_d_/SnO_2_, Ana-SnO_2_ to hydrothermal assembled A_d_-SnO_2_. By looking into the performance of Ana-SnO_2_-PGN and SnO_2_[O]rTiO_2_-PGN (Fig. 4c and Supplementary Fig. S8c), we conclude that the 200–300 capacity increment in SnO_2_[O]rTiO_2_-PGN is attributed to the advantage of A_d_ rTiO_2,_ which can easily form Ti–O–Sn bonding through condensation between Ti–OH and Sn–OH and further steadily hold SnO_2_ to maintain high reversible capacity. Besides, another ternary-phase control experiment, A_d_-SnO_2_-G, is utilized to indicate the essential role of PGN in cushioning SnO_2_ volume expansion and the active electrochemical absorption contribution. The replacement of conventional reduced graphene oxide layers to the PGN buffering membrane can realize significant performance enhancement, as the dark cyan circle and blue rhombus capacity profiles are shown in Fig. 4c. The A_d_/SnO_2_-PGN sample (physically mixed A_d_ and SnO_2_ in PGN) in control group C only realizes around 300 mAh g^-1^ after 100 cycles, 200 mAh g^−1^ lower than SnO_2_[O]rTiO_2_-PGN configuration. The comparison again implies the importance of chemical bonding (Ti–O–Sn and Ti–O–C) interconnections in the SnO_2_[O]rTiO_2_-PGN design. The discharge profiles paired with charging profiles in Fig. 4a–c are shown in Supplementary Fig. S8a–c. Moreover, we conduct the optimization process to find the appropriate recipe by adjusting the A_d_ rTiO_2_ and SnO_2_ mass ratio (as summarized in Supplementary Table S5). The XRD characteristic peak intensity ratios among peak a, 26.6° of SnO_2_ and b, 47.6° of rTiO_2_ increase with the continuous addition of SnO_2_ in SnO_2_[O]rTiO_2_-PGN, as indicated in Supplementary Fig. S3d and Supplementary Table S5. The GCD profiles in Supplementary Fig. S8d,e reveal that the higher percentage of SnO_2_ in the SnO_2_[O]rTiO_2_-PGN exhibits enhanced capacity in the initial 20 cycles but then decays during the following cycles. Finally, the SnO_2_[O]rTiO_2_-PGN with 6:3 (2:1) rTiO_2_ and SnO_2_ ratio attain the highest reversible capacity (540 mAh g^−1^ until 120 cycles).

To trace the electrochemical process, we present the charge–discharge (CD) profiles and cyclic voltammetry (CV) measurements in Fig. 4d–g. The CD process of SnO_2_[O]rTiO_2_-PGN has three turning points at around 0.2, 0.5, and 2.0 V in the charging process and two plateau regions at around 1.8 and 0.1 V, which are highly consistent with the CV redox peaks. As shown in the CV scan of SnO_2_[O]rTiO_2_-PGN in Fig. 4e, the cathodic peak at 1.75 V and anodic peak at 2.01 V can be assigned to reversible Li^+^ intercalation/deintercalation in rTiO_2_^15^. The cathodic peaks at 0.12 and 0.95 V and anodic peaks at 0.15, 0.53 and 1.26 V are attributed to reversible alloying/de-alloying and the partial conversion reaction of Li^+^ and SnO_2_^20^. In Fig. 4d, we can observe that SnO_2_[O]rTiO_2_-PGN profile slopes at near 0.1 V of the discharging process and near 0.5 V of the charging process are smaller than A_d_-SnO_2_-G and Ana-SnO_2_-PGN, indicating more energy storage activities of SnO_2_ are preserved in SnO_2_[O]rTiO_2_-PGN. The first cycle CD and CV results and the further CV cycling curves (6th to 10th cycle) of SnO_2_[O]rTiO_2_-PGN are supplied in Supplementary Fig. S9 as supporting information. The early CV curves until the 10th cycles show almost identical shape (Supplementary Fig. S9c), and the further mid- and late-CV cycling from 30th to 80 cycles present similar trends with slight changes during cycling (Supplementary Fig. S9d). And the dQ/dV plots of early- (a, 10th cycle), mid- (b, 100th cycle), and late-cycling (c, 200th cycle) also indicate further cycling of SnO_2_[O]rTiO_2_-PGN anode keep the similar lithiation/delithiation behavior among early-, mid- and late-cycles (Supplementary Fig. S10).

To obtain the energy storage performance under different conditions, we perform rate capability (RC) measurements in a 20-fold increment current density range among SnO_2_[O]rTiO_2_-PGN, A_d_-SnO_2_-G, Ana-SnO_2_-PGN, and Ana. TiO_2_ anodes. As presented in the RC charging capacity profiles shown in Fig. 4f, the SnO_2_[O]rTiO_2_-PGN LIB anode reaches around 530, 480, 450, 424, 390, 350 mAh g^−1^ under 10 to 200 mA g^−1^ respectively, and back 550 mAh g^−1^ after return to 10 mA g^−1^, which indicates the high energy storage capability and reversibility under various conditions. Moreover, specific capacities of A_d_-SnO_2_-G and Ana-SnO_2_-PGN are lower than SnO_2_[O]rTiO_2_-PGN under all the investigated current density ranges, following the GCD cycling performance sequence. The A_d_-SnO_2_-G and Ana-SnO_2_-PGN exhibit rapid capacity decay under the first analyzing condition of 10 mA g^−1^. The SnO_2_[O]rTiO_2_-PGN electrode shows capacity fading in the first five cycles and stabilizes in the subsequent cycles. The phenomena expressed by the several initial cycles are thought to be due to a few isolated SnO_2_ nanoparticles species that inevitably unbonded with rTiO_2_ and PGN. After several initial cycles, SnO_2_[O]rTiO_2_-PGN presents a stable cycling character under varied current densities. The single-phase control group sample, Ana. TiO_2_ reveals about 50/40/33/26/18/14/40 mAh g^−1^ Li^+^ storage capacity at 10/20/30/50/100/200/10 mA g^−1^, respectively. The SnO_2_[O]rTiO_2_-PGN LIB anode reaches 100.6/12.0/13.6/16.3/21.6/25.0/13.8 times higher specific capacity than the conventional pristine Ana. TiO_2,_ at various conditions, demonstrating its promising energy storage property. The discharge profiles of RC measurements are shown in Supplementary Fig. S11, which also follow the RC charging performance trend and phenomena. We conduct long-term cycling tests to confirm the performance superiority and electrode stability of the SnO_2_[O]rTiO_2_-PGN anode (Fig. 4g). The SnO_2_[O]rTiO_2_-PGN anode preserves 600 mAh g^−1^ capacity after 250 cycles under 100 mA g^−1^ and reveals high reversibility with 100% CE after passing several initial cycles. It was reported that the investigating anode capacity decayed seriously from around 250th cycles without switching the Li metal counter electrode^43^. We refresh the cell with the new Li foil counter electrode, electrolyte, and separator after the 100th cycle to prevent the degradation of the Li metal counter electrodes affecting the cyclability of the SnO_2_[O]rTiO_2_-PGN^44^. Moreover, we compare the designed SnO_2_[O]rTiO_2_-PGN anode with the current commercialized graphite LIB anode and observe that the specific capacity of SnO_2_[O]rTiO_2_-PGN is about 330 mAh g^−1^ higher, or about two-folds that of graphite (around 270 mAh g^−1^ after 250 cycles of graphite anode) after long-term cycling (Supplementary Fig. S12). Additionally, we summarized the achieved capacities of SnO_2_, TiO_2,_ and their composites in literature (Supplementary Table S6). This observation further supports SnO_2_[O]rTiO_2_-PGN as a promising alternative to the conventional graphite of the most widely applied LIB anode.

### Energy storage behavior kinetics and cushioning protection

**Figure 5:**
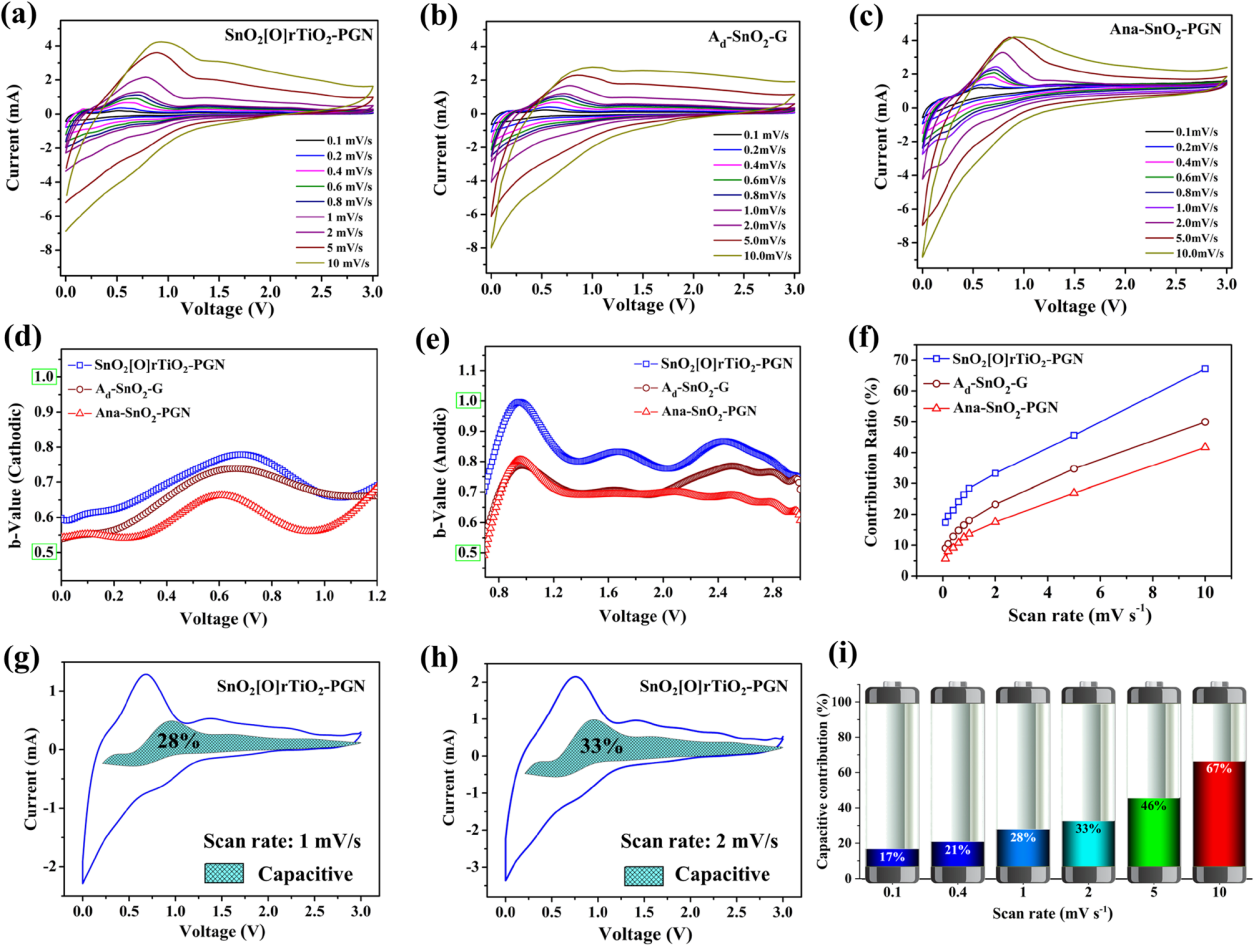
Energy storage behavior kinetics analysis. (**a**–**c**) CV scans of the SnO_2_[O]rTiO_2_-PGN, A_d_-SnO_2_-G, and Ana-SnO_2_-PGN anodes, respectively, at various speeds (0.1–10 mV s^−1^). (**d**,**e**) Plots of b-value versus voltage of cathodic and anodic scan, respectively. (**f**) Pseudo-capacitive contribution ratio (indicated as “contribution ratio, %”) under various scan rates. (**g**,**h**) The separation of capacitive and diffusion-controlled process contributions under 1 and 2 mV s^−1^. (**i**) Normalized contribution ratio bar plot of SnO_2_[O]rTiO_2_-PGN under various scan rates.

To obtain an in-depth understanding of the energy storage behaviors, we carry out the CV scans of the SnO_2_[O]rTiO_2_-PGN, A_d_-SnO_2_-G, and Ana-SnO_2_-PGN anodes under various speeds (0.1–10 mV s^−1^), as shown in Fig. 5a–c. The purpose of analyzing the A_d_-SnO_2_-G anode is to demonstrate the influences of electrochemical kinetics from the PGN phase. Similarly, we intend to reveal the contribution of A_d_ rTiO_2_ in SnO_2_[O]rTiO_2_-PGN by comparing the electrochemical behavior of SnO_2_[O]rTiO_2_-PGN and Ana-SnO_2_-PGN. As the scan rate increases from 0.1 to 10 mV s^−1^, the shapes of each sample’s CV patterns are similar and exhibit broad lithiation and delithiation peaks. Based on the expression of the power law, the current response (i, mA) of the CV scan correlates with the value of scan rate (*v*, mV s^−1^) and follows the specific formula, as shown in Eq. (1):^45^

1 $$i=a{v}^{b}.$$

We can transform Eqs. (1) to (2) after taking the logarithm:

2 $$\mathrm{log}(i)=b\mathrm{log}\left(v\right)+\mathrm{log}(a),$$

where *a* is a constant and b can be derived from the slope of log (*i*) versus log (*v*). There are two specific boundary values of b: *b* = 0.5 and *b* = 1^46^. In the case of *b* = 0.5, the current is proportionate to the square root of scan rate, *v*. The diffusion-controlled lithium storage process is considered when the *b* value is equal or close to 0.5. Another well-defined condition, *b* = 1, typically indicates the surface-controlled capacitive contribution according to the proportional relationship of capacitive current with sweeping rate. By looking through the CV scan files of the three samples in Fig. 5a–c, we can observe that the main electrochemical reactions of cathodic scan (3–0 V) are in the range of 1–0 V, and the anodic reactions are dominant in 0.7–3.0 V. Therefore, we calculate and plot the *b*-value versus V of cathodic scan (1.2–0 V) and anodic scan (0.7–3 V) to analyze the energy storage mechanism and electrochemical processes of SnO_2_[O]rTiO_2_-PGN (Fig. 5d,e). The b-values of the cathodic scan in the active electrochemical range follow the order of SnO_2_[O]rTiO_2_-PGN > A_d_-SnO_2_-G > Ana-SnO_2_-PGN (Fig. 5d). The cathodic b-value profiles of all three samples display bumps that are located in the range 0.5–0.9 V, and approach the surface control capacitive process boundary. These bumps may originate from the lithiation reaction of SnO_2_ nanoparticles and Li^+^ storage on the phase interfaces, giving higher-level capacitive contribution. As shown in Fig. 5e, the anodic *b*-values also exhibited the same trend of SnO_2_[O]rTiO_2_-PGN > A_d_-SnO_2_-G > Ana-SnO_2_-PGN in the active range of 0.7–3.0 V. Moreover, the *b*-values of SnO_2_[O]rTiO_2_-PGN show three peaks with higher *b*-value than the control group sample at 0.9, 1.7, and 2.5, respectively. The *b*-value peaks at 0.9 and 1.7 V are supposed from the delithiation process of SnO_2_ nanostructures and Li^+^ extraction from phase boundaries, which can pull the b values to the near surface-controlled process. The differentiation of anodic b-values among the three samples in the range of 2–3 V may result from the PGN and A_d_ rTiO_2_ surface capacitive processes, which give SnO_2_[O]rTiO_2_-PGN a higher b-value than A_d_-SnO_2_-G (PGN contribution), and Ana-SnO_2_-PGN (A_d_ rTiO_2_ contribution).

Based on the above-calculated b values, we can deduce that the pseudo-capacitive process from surface-controlled capacitive contribution coexists with the diffusion-controlled intercalation process in the SnO_2_[O]rTiO_2_-PGN anode. To study the origins of SnO_2_[O]rTiO_2_-PGN anode advancements, we quantitatively analyze the pseudo-capacitive contribution percentages in the overall reversible capacity among the three anode samples (SnO_2_[O]rTiO_2_-PGN, A_d_-SnO_2_-G, and Ana-SnO_2_-PGN). By analyzing the relationship between current and CV scan rate under a fixed potential value, we can successfully separate the two types of energy storage processes according to Eqs. (3) and (4):^46^

3 $$i(V)={{k}_{1}v+k}_{2}{v}^{1/2},$$

4 $$i(V)/{v}^{1/2}={{k}_{1}{v}^{1/2}+k}_{2},$$

where *k*_1_ and *k*_2_ are slope and intercept in Eq. (4). We can differentiate the current responses from the ${k}_{1}v$ (surface-controlled pseudocapacitive process) and ${k}_{2}{v}^{1/2}$ (diffusion-controlled intercalation processes). After the data processing based on the equations, we acquire the pseudo-capacitive contribution ratio (simplified as “contribution ratio, %” in the following discussion) under various scan rates and plot them in Fig. 5f. We notice that the contribution ratio of SnO_2_[O]rTiO_2_-PGN is always higher than those of the A_d_-SnO_2_-G and Ana-SnO_2_-PGN control samples, which also follows the same GCD performance order of the three samples. Introducing the surface-controlled pseudocapacitive process in the SnO_2_[O]rTiO_2_-PGN plays an essential role in realizing the high capacity. The SnO_2_[O]rTiO_2_-PGN anode exhibits a pseudocapacitive contribution that is around 16% and 26% higher than those of Ana-SnO_2_-PGN at 2 and 5 mV s^−1^, respectively. The A_d_-SnO_2_-G construction reveals contribution ratios that are 10% and 17% lower than SnO_2_[O]rTiO_2_-PGN. These results support demonstrating the covalent bonding formation between the species (rTiO_2_, SnO_2_ and PGN), and the existence of large interlayer spacing active PGN can effectively create the interfaces and prevent nanoparticle aggregation and expose more active surface, finally giving a higher portion of pseudo-capacitive energy storage behavior. We display the separation of capacitive and diffusion current and the integrated pseudo-capacitance area in Fig. 5g,h for SnO_2_[O]rTiO_2_-PGN and Figure S13 for A_d_-SnO_2_-G and Ana-SnO_2_-PGN control samples. The shapes of the SnO_2_[O]rTiO_2_-PGN integrated pseudo-capacitance area at 1 and 2 mV s^−1^ reveal the downward extension in a 0.5–0.8 V and upward bump near 0.9 V coincident with the higher b-value range in the cathodic and anodic scan, respectively. Under most investigated sweep conditions, the pseudo-capacitive process contributions are less than the diffusion-controlled intercalation and alloying processes from 0.1 to 5 mV s^−1^, as shown in Fig. 5i of normalized contribution ratio. It indicates that the energy storage processes of the SnO_2_[O]rTiO_2_-PGN anode are dominated by diffusion-controlled behavior but present an increased surface-controlled pseudo-capacitive percentage relative to the other control group configurations. The mechanism investigation diagram based on experimental design are illustrated in Supplementary Fig. S14.

**Figure 6:**
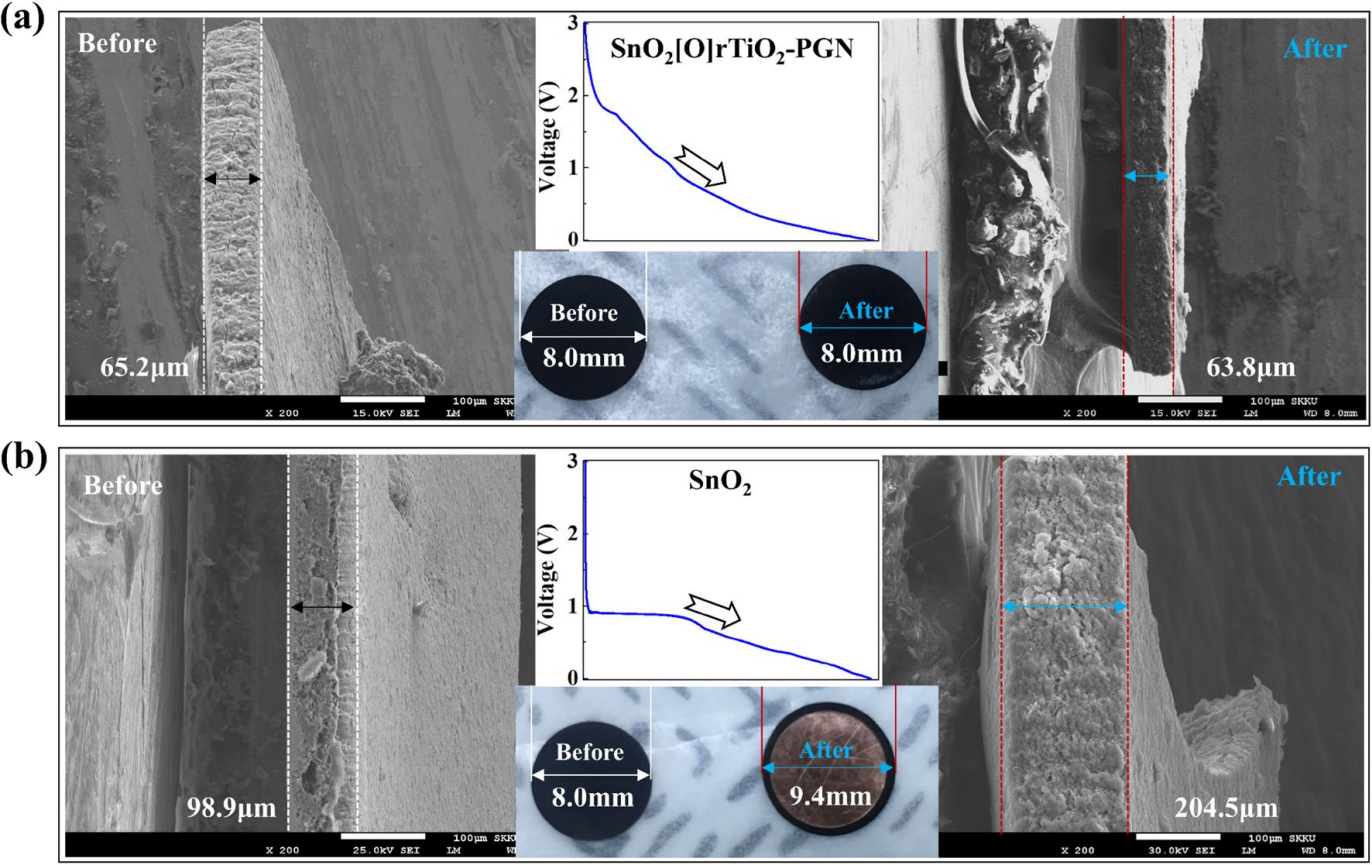
Post-analysis of the lithiated electrodes. Electrode images (**a**) before and (**b**) after lithiation of SnO_2_[O]rTiO_2_-PGN and SnO_2_. The associated voltage vs. time profile is inserted in the middle. Additionally, the measured size information is noted in the figures.

To demonstrate the sufficient cushioning capability of the SnO_2_[O]rTiO_2_-PGN structure, we perform the post-analysis of the lithiated SnO_2_[O]rTiO_2_-PGN and SnO_2_ anodes to investigate the electrode volume variation (Fig. 6). After full lithiation, SnO_2_[O]rTiO_2_-PGN film displays unchanged electrode thickness and diameter (Fig. 6a) and reveals robust mechanical stability due to its unique cushioning design and steady chemical bonding interconnections. The SnO_2_ electrode film shows noticeable volume expansion after the lithiation process, with the lithiated SnO_2_ electrode volume reaching around 285% of the initial volume as calculated based on the observed dimension variation (Fig. 6b). The serious volume expansion possesses severely detrimental influences on battery performance and operation safety by inducing electrode pulverization, active material detachment from the current collector, and battery swelling^47^. The successful management of electrode volume change by the SnO_2_[O]rTiO_2_-PGN configuration can achieve promising active electrode materials' performance advancements and give reliable battery system security.

## Conclusions

In this work, we proposed a SnO_2_[O]rTiO_2_-PGN configuration and successfully sutured the chemically bonded metal oxides (disordered rTiO_2_ and SnO_2_) into a versatile cushioning graphite network in energy storage. Various characterization approaches were implemented to provide evidence of covalent chemical bonding between phases in the composite. We also revealed the microstructures of SnO_2_[O]rTiO_2_-PGN construction that SnO_2_ nanoparticles discretely surrounded on A_d_ rTiO_2_ and wrapped by the ~ 1.2 nm interlayer distance cushioning PGN buffer membrane. The built-in unique PGN system and steady bonding interconnection between species of SnO_2_[O]rTiO_2_-PGN can contribute to protecting electrode integrity under significant volume variation situations and retaining high performance and long-term reversibility. We further confirmed the energy storage superiority of the SnO_2_[O]rTiO_2_-PGN configuration by comparison with the other 16 control group samples (Single, Binary, and Ternary phases combination). The SnO_2_[O]rTiO_2_-PGN concept and structure suggested in this study provide an efficient approach for tackling serious electrode performance fading and enhancing energy storage capability, which could stimulate the structural design of electrode composites with sufficient volume stability.

## Supplementary Information

Supplementary Information.

## Data Availability

The datasets generated and/or analyzed during the current study are not publicly available due to the confidential policy of the research center but are available from the corresponding author on reasonable request.
