# Supplementary material for: Pseudo-capacitive and kinetic enhancement of metal oxides and pillared graphite composite for stabilizing battery anodes
Source: Sci Rep. 2022 Jul 15;12:12079. doi: 10.1038/s41598-022-15789-0 (PMC9287451; doi:10.1038/s41598-022-15789-0)
Supplement: Supplementary file 1 — Supplementary Information. [file 41598_2022_15789_MOESM1_ESM.docx]

Supporting Information

Pseudo-capacitive and Kinetic Enhancement of Metal Oxides and Pillared Graphite Composite for Stabilizing Battery Anodes

Yongguang Luo,^bf^ Lingling Wang,^ab^ Qian Li,^c^ Jungsue Choi,^ab^ G. Hwan Park,^ab^ Zhiyong Zheng,^b^ Yang Liu,^ab^ Hongdan Wang,^ab^ Hyoyoung Lee, *^abde^

^a^ Center for Integrated Nanostructure Physics (CINAP), Institute for Basic Science (IBS), 2066 Seoburo, Jangan-gu, Suwon 16419, Republic of Korea.

^b^ Department of Chemistry, Sungkyunkwan University, 2066 Seoburo, Jangan-gu, Suwon 16419, Republic of Korea.

^c^ Department of Applied Environmental Science, College of Engineering, Kyunghee University, Yongin, 17104, Republic of Korea.

^d^ Department of Biophysics, Sungkyunkwan University, 2066 Seoburo, Jangan-gu, Suwon 16419, Republic of Korea.

^e^ Creative Research Institute, Sungkyunkwan University, 2066 Seoburo, Jangan-gu, Suwon 16419, Republic of Korea.

^f^ BYD Company Ltd., 1301 Shenshan Road, Pingshan District, Shenzhen 518122, China

* Correspondence and requests should be addressed to H.L. ([hyoyoung@skku.edu](mailto:hyoyoung@skku.edu))

Supporting Figure S1-S14

Supporting Table S1-S6


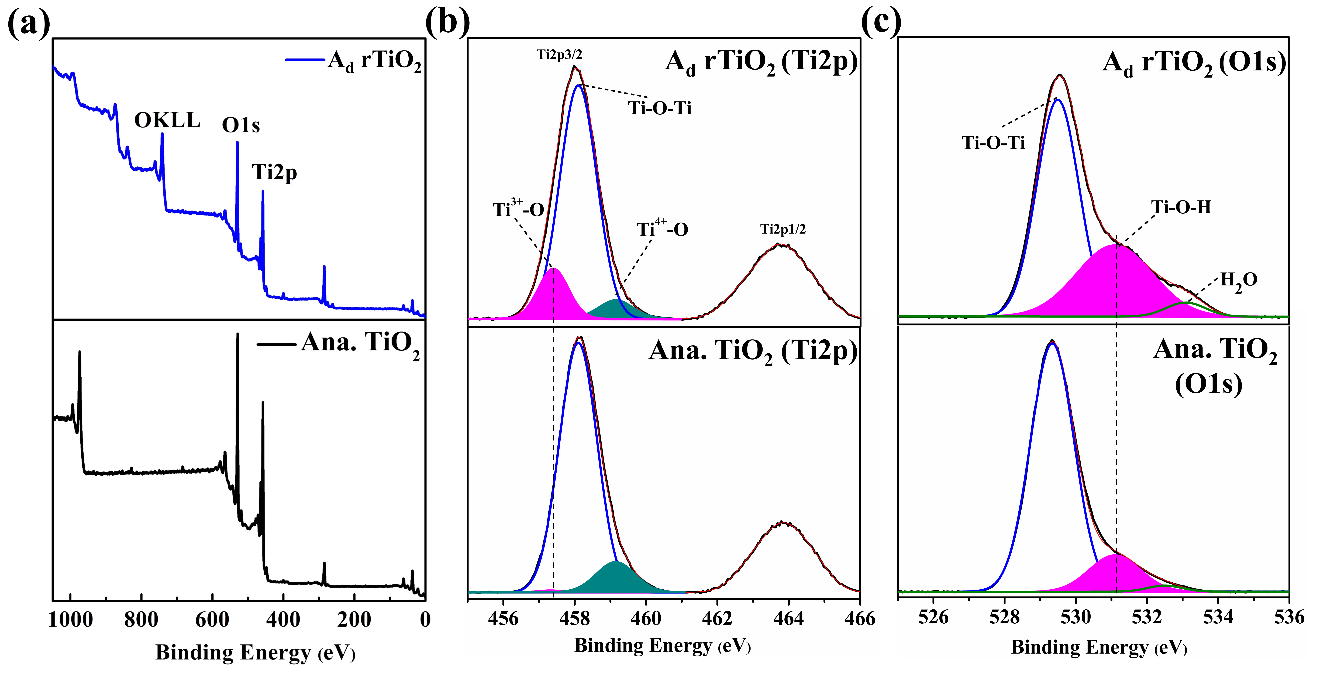


**Figure S1**. XPS of A_d_ and Ana. TiO_2_. (a) represents the full spectra survey. (b) shows the Ti2p spectra. The Ti^3+^ portion is increased in A_d_ rTiO_2_ according to the deconvoluted peaks. Ti^4+^-O stands for the dangling bond in the edge of the nanostructure. (c) are the O1s spectra. Ti-OH percentage is enhanced, which attributes from the rTiO_2_.


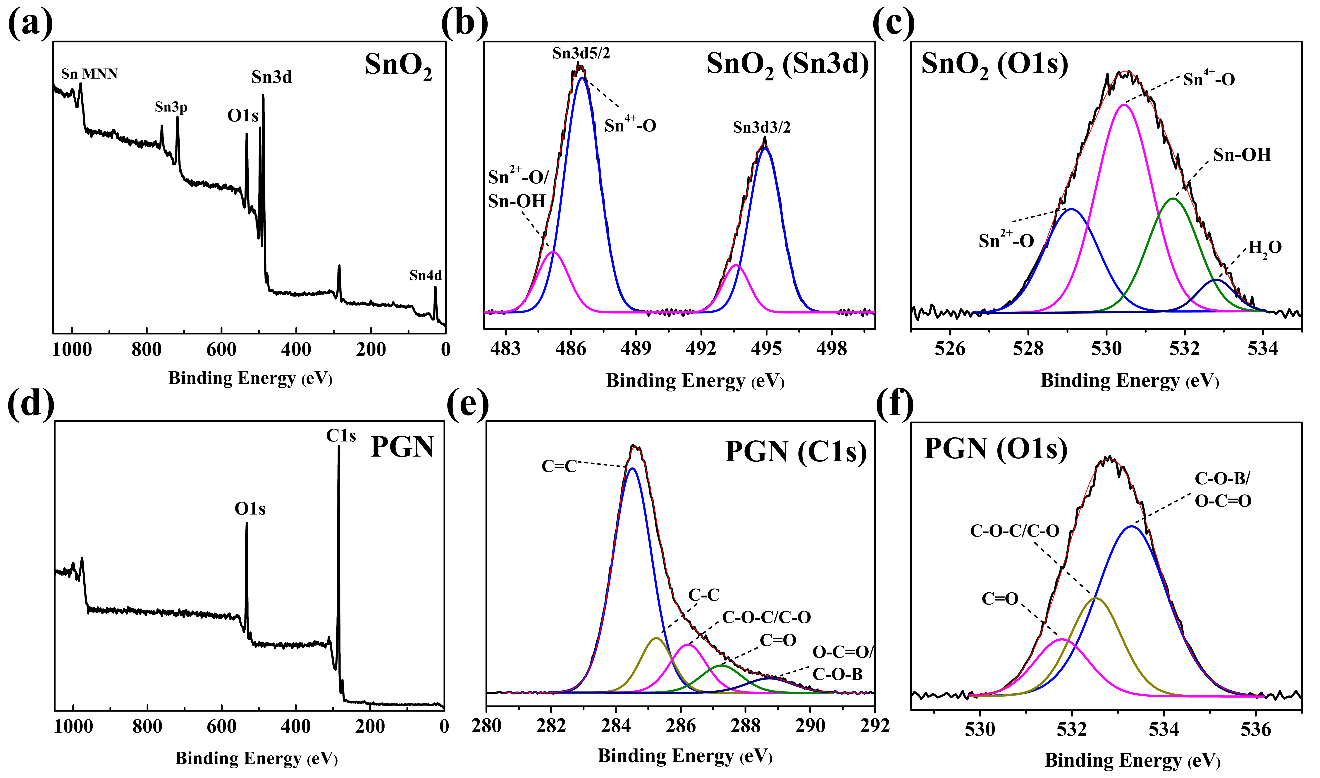


**Figure S2**. XPS of synthesized SnO_2_ and PGN. (a) and (d) represent the full survey spectra of SnO_2_ and PGN, respectively. (b) and (c) show the Sn3d and O1s of SnO_2_. (e) and (f) are the C1s and O1s of PGN.


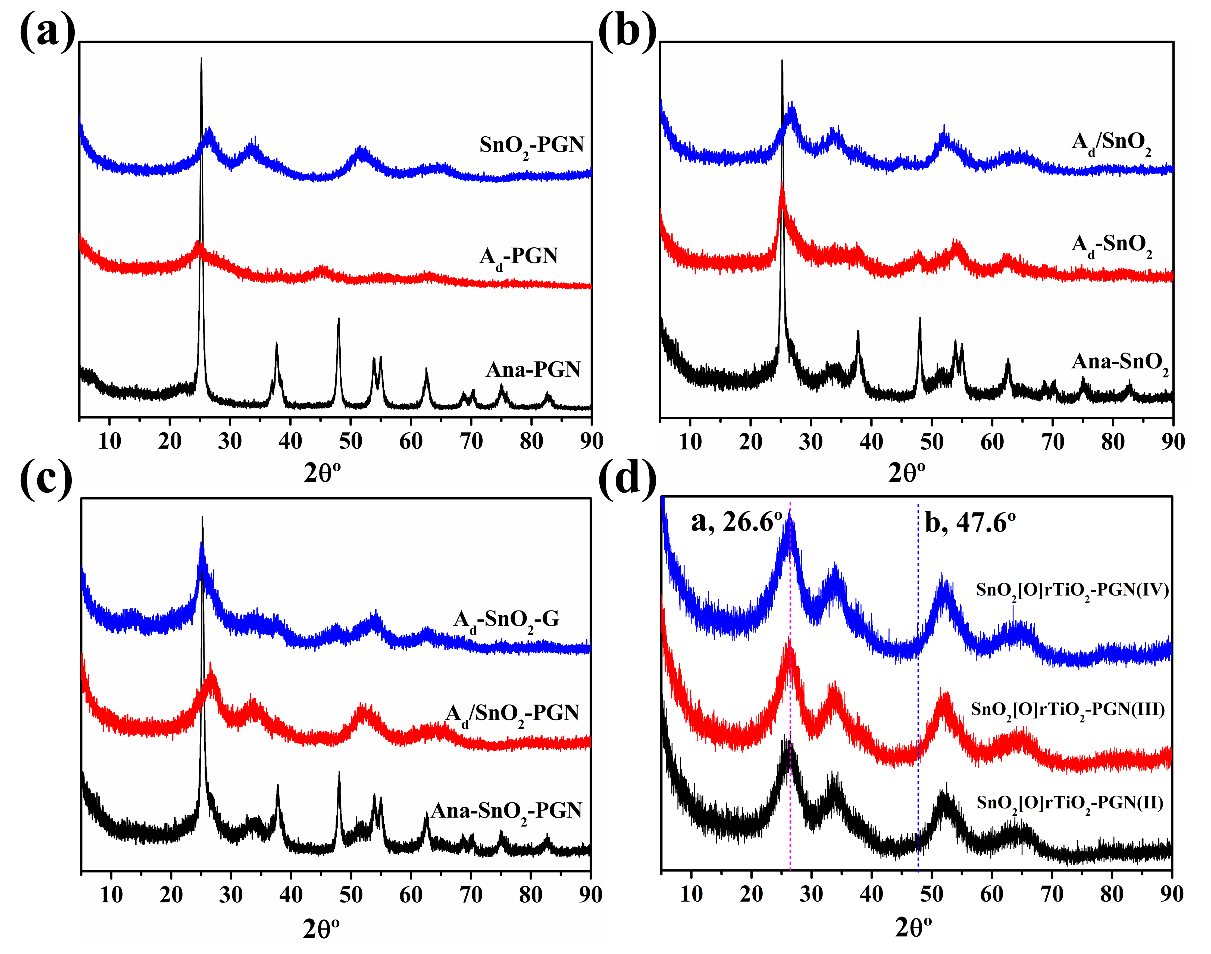


**Figure S3**. The XRD spectra of control group samples. (a) for Group A, Single-phase samples; (b) for Group B, Binary-phases samples; (c) for Group C, Ternary-phases samples; And (d) for the SnO_2_[O]rTiO_2_-PGN with different ratio of the A_d_ rTiO_2_ and SnO_2_.


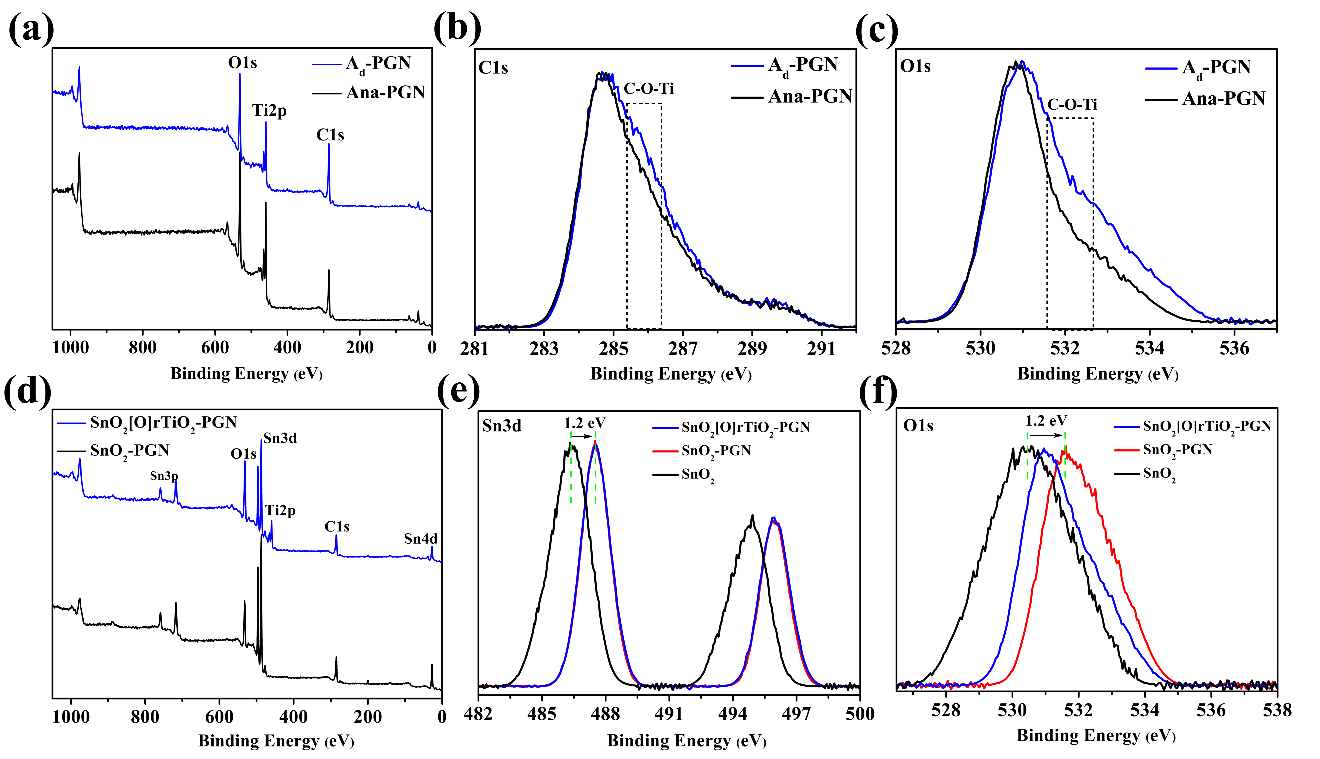


**Figure S4**. XPS spectra comparison of SnO_2_[O]rTiO_2_-PGN with Binary-phases control group samples. (a) and (d) represent the full survey spectra of A_d_-PGN & Ana-PGN and SnO_2_[O]rTiO_2_-PGN & SnO_2_-PGN, respectively. (b) and (c) show the C1s and O1s of A_d_-PGN & Ana-PGN, which note the C-O-Ti region in the figures. (e) and (f) are the Sn3d and O1s of SnO_2_[O]rTiO_2_-PGN, SnO_2_-PGN and SnO_2_. The peak shifting is observed due to the changes in the bonding states.


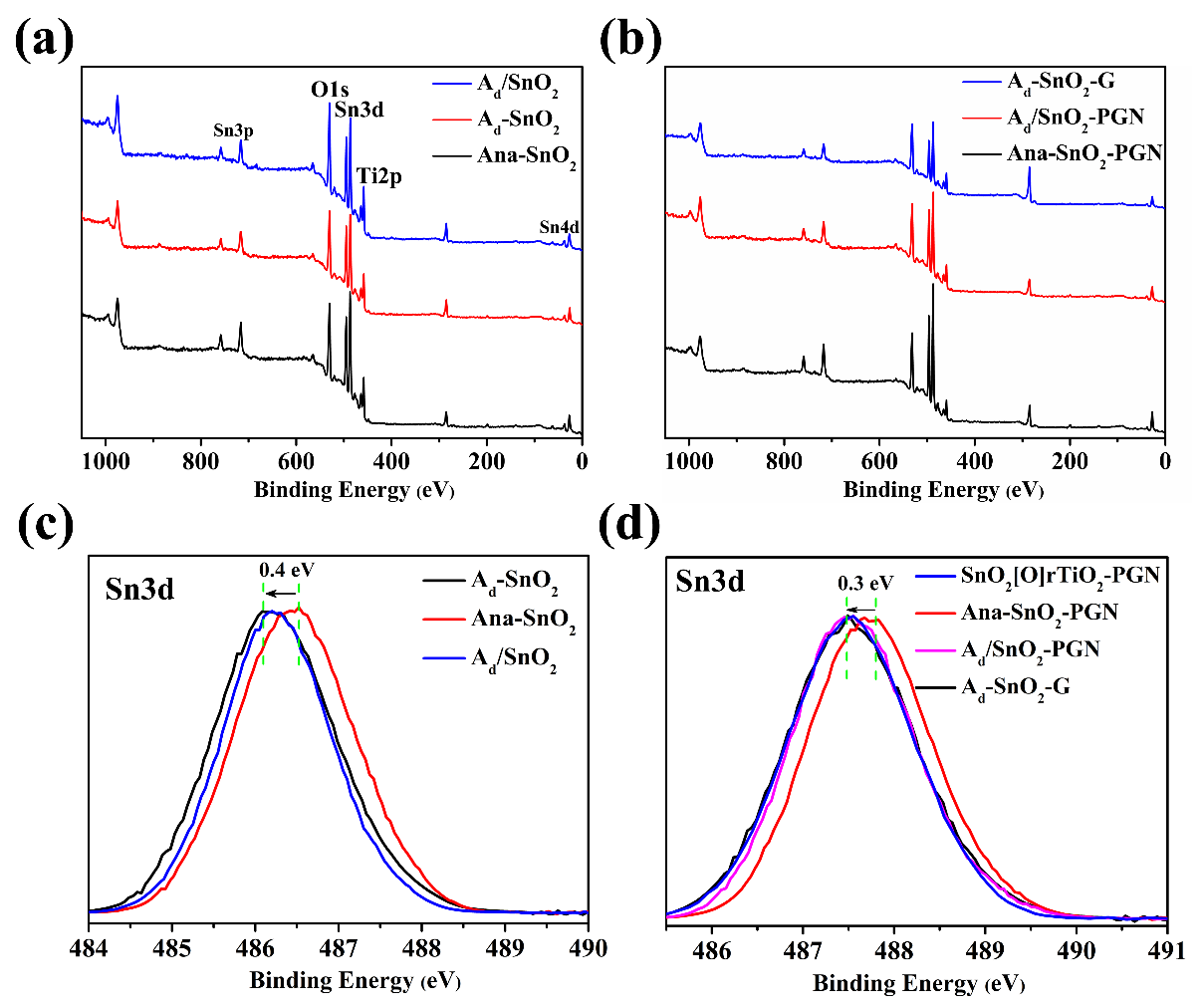


**Figure S5**. XPS spectra comparison of Binary-phases and Ternary-phases control group samples. (a) and (b) represent the full survey spectra of A_d_/SnO_2_, A_d_-SnO_2_ & Ana-SnO_2,_ and A_d_-SnO_2_-G, A_d_/SnO_2_-PGN & Ana-SnO_2_-PGN, respectively. (c) and (d) present the Sn3d peaks shifting among the A_d_/SnO_2_, A_d_-SnO_2_ & Ana-SnO_2_ and SnO_2_[O]rTiO_2_-PGN, A_d_-SnO_2_-G, A_d_/SnO_2_-PGN & Ana-SnO_2_-PGN, respectively.


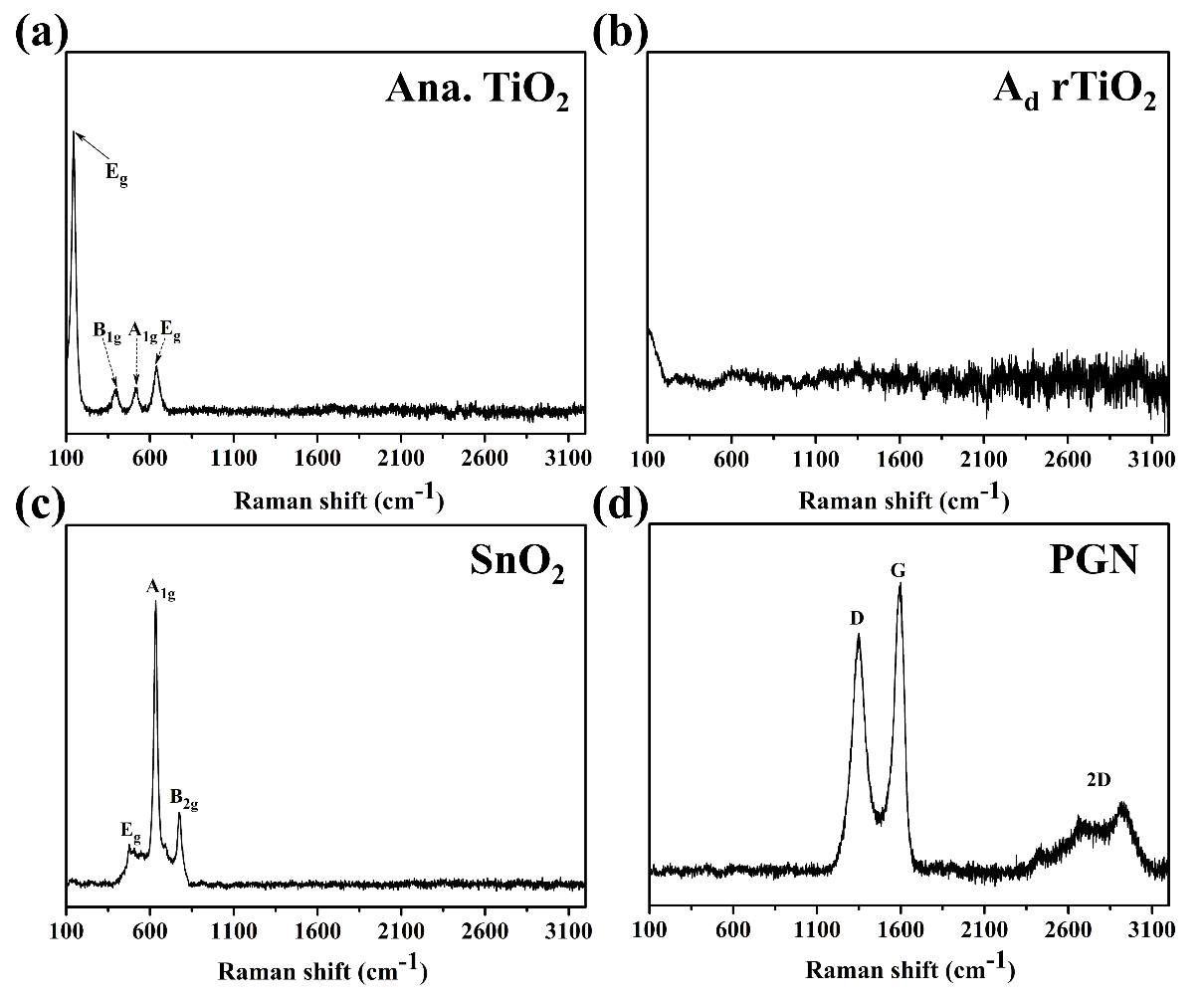


**Figure S6**. Raman spectrum of the single component in SnO_2_[O]rTiO_2_-PGN. (a) for Ana.TiO_2_, (b) for A_d_ rTiO_2_, (c) for synthesized SnO_2_ and (d) for PGN.


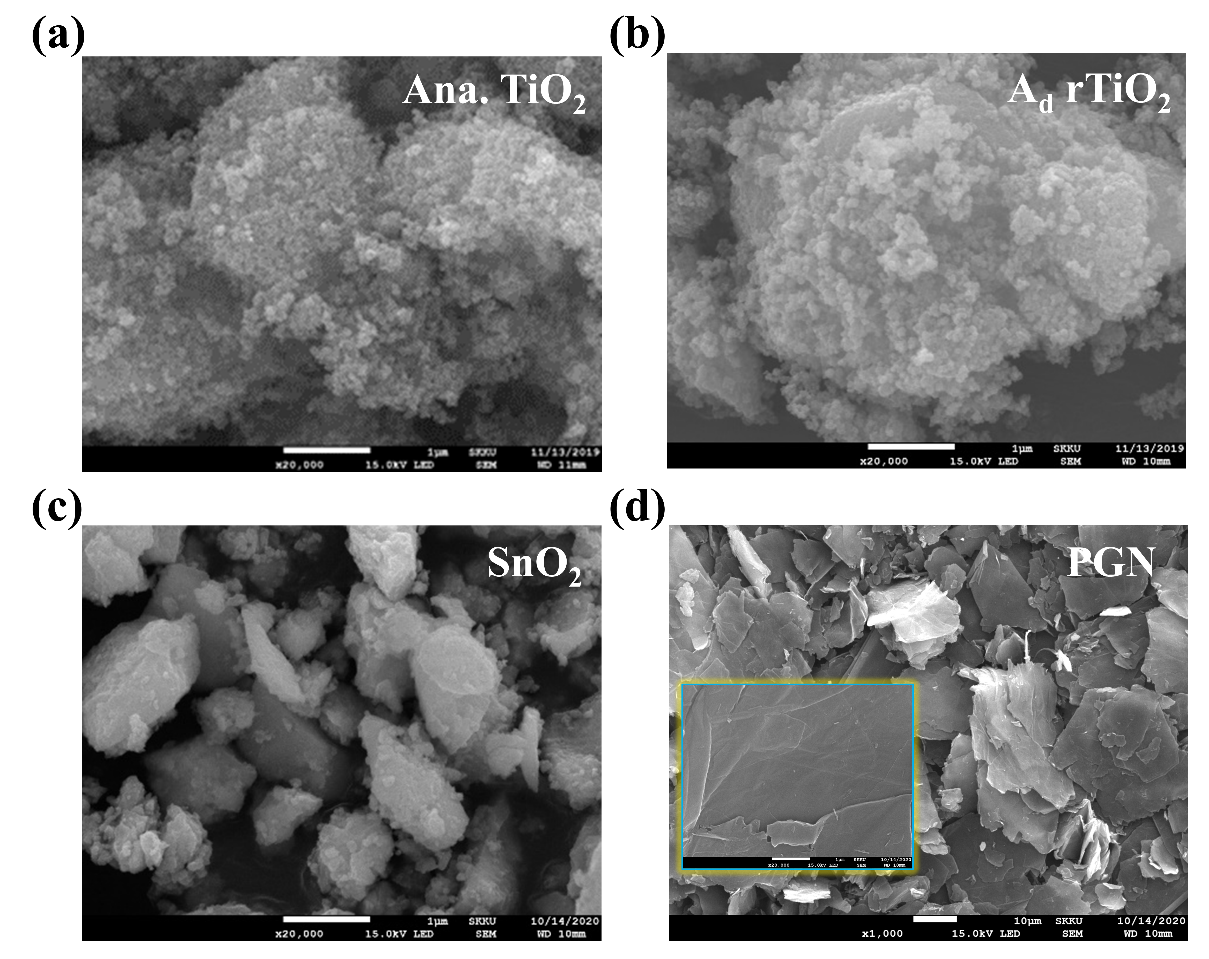


**Figure S7.** SEM images of every single phase in SnO_2_[O]rTiO_2_-PGN. (a) for Ana.TiO_2_, (b) for A_d_ rTiO_2_, (c) for synthesized SnO_2_ and (d) for PGN. And the magnified image on the surface of one PGN sheet is inserted into the (d).


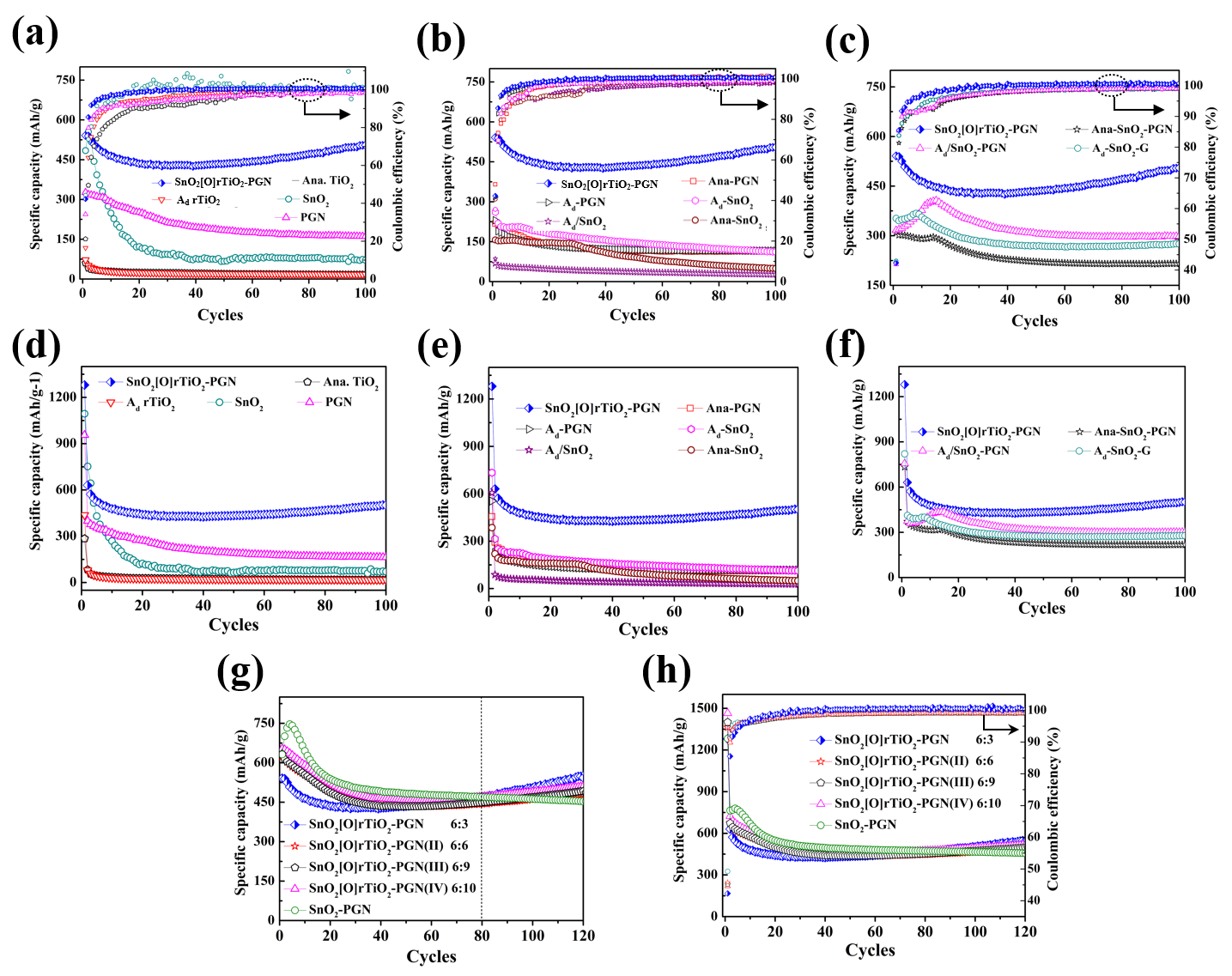


**Figure S8.** Electrochemical performance investigation. (a), (b) and (c) present the cycling performance comparisons among SnO_2_[O]rTiO_2_-PGN with all of the single/binary/ternary phase samples under 100 mA g^-1^; (d), (e), and (f) show the discharge profiles paired with charging profiles in Figure 3 a-c. (g) and (h) present the charge and discharge profiles of the SnO_2_[O]rTiO_2_-PGN, SnO_2_[O]rTiO_2_-PGN(II-IV), and SnO_2_-PGN to show performance changes with different rTiO_2_ and SnO_2_ ratio.

**
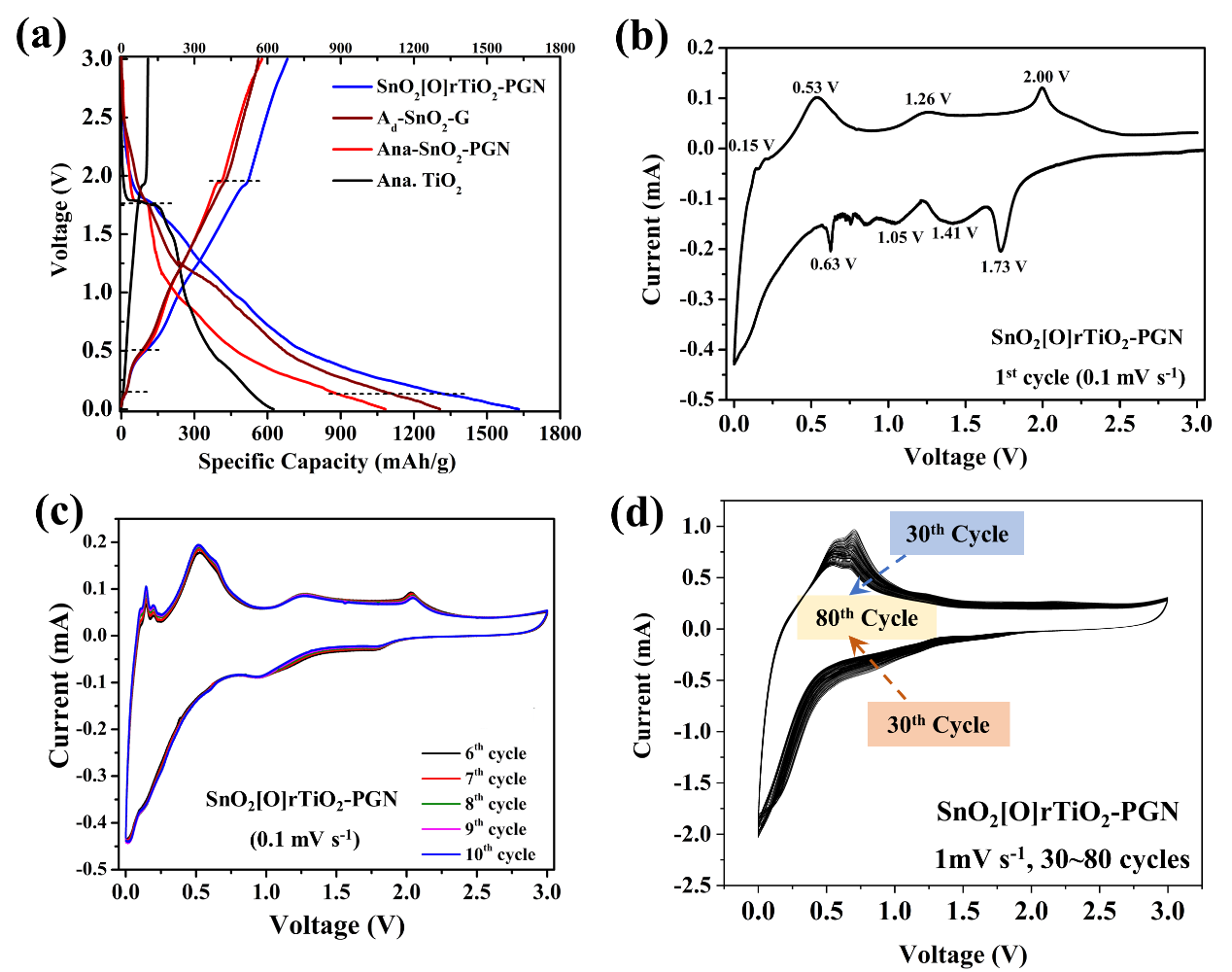
**

**Figure S9.** (a) shows the first cycle CD profiles of SnO_2_[O]rTiO_2_-PGN, A_d_-SnO_2_-G, Ana-SnO_2_-PGN, and Ana. TiO_2_. (b) and (c) represents the first and 6^th^ to 10^th^ cycles of CV measurements under 0.1 mV s^-1^ for SnO_2_[O]rTiO_2_-PGN. (d) shows the CV curves from 30^th^ cycle to 80^th^ cycle for the comparison among early cycling, mid cycling, and late cycling.

**
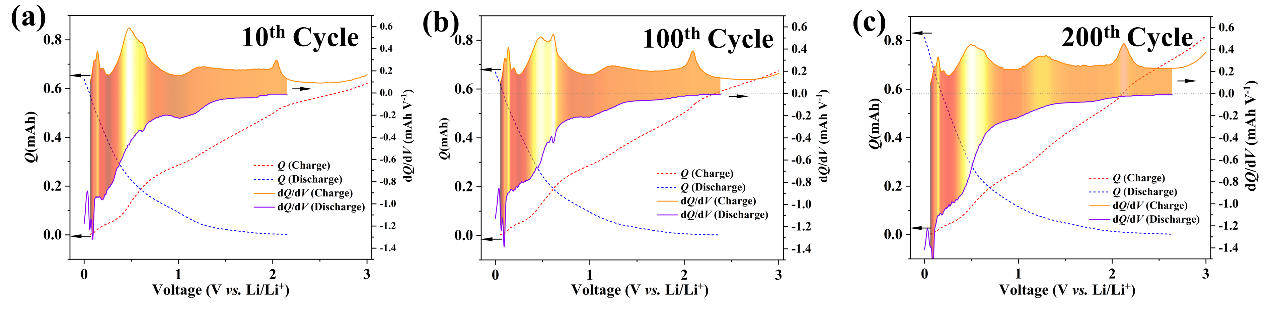
**

**Figure S10.** The dQ/dV plots of early- (a, 10^th^ cycle), mid- (b, 100^th^ cycle), and late-cycling (c, 200^th^ cycle).

**

**

**Figure S11.** The discharge profiles of RC measurements range from 10 to 200 mA g-1 and return to 10 mA g^-1^.

**

**

**Figure S12.** The long-term cycling of SnO_2_[O]rTiO_2_-PGN and graphite LIB anode under 100 mA g^-1^.


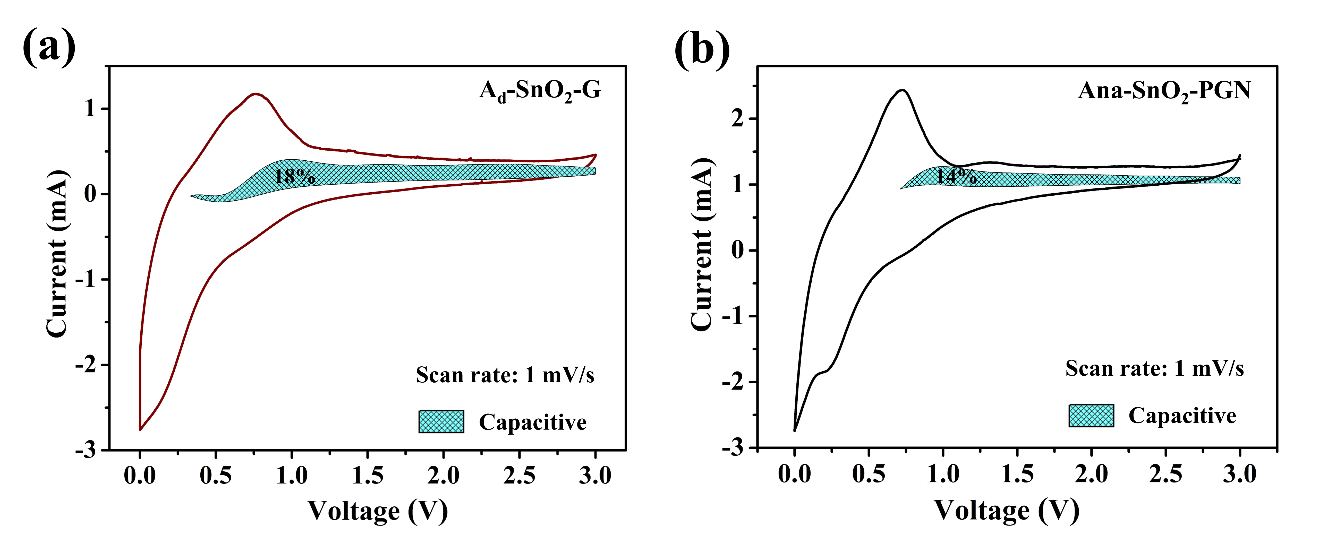


**Figure S13.** The separation of capacitive and diffusion current and the integrated pseudo-capacitance area of A_d_-SnO_2_-G and Ana-SnO_2_-PGN control samples under 1 mV s^-1^.

**
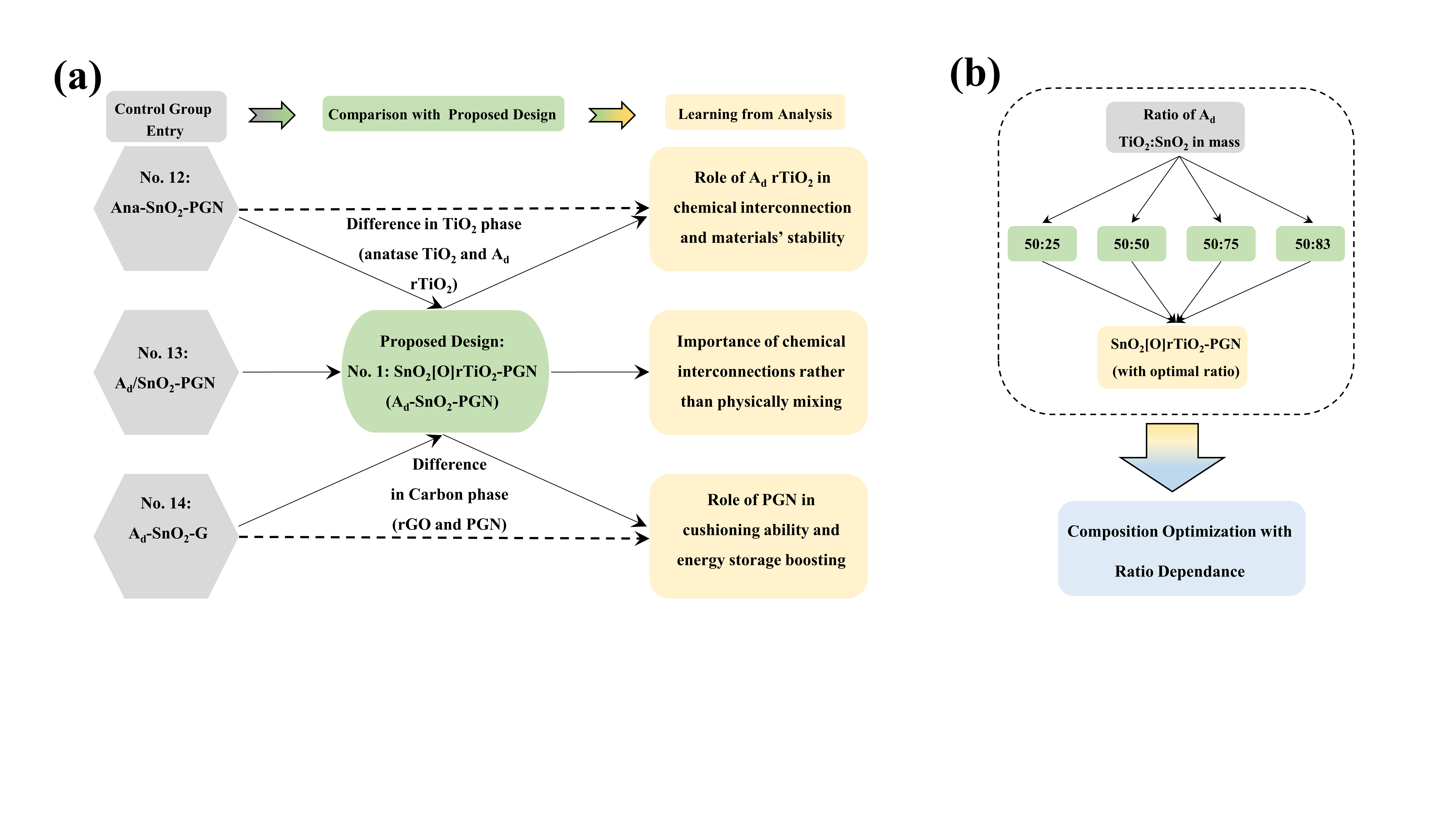
**

**Figure S14.** The mechanism investigation diagram based on experimental design. a) the comparison among samples. b) optimization for proper metal oxides ratio with ratio dependance investigation.

**Table S1.** The percentage of the deconvoluted species in anatase TiO_2_ and A_d_ rTiO_2_ samples.

| Deconvoluted species | | Ti^3+^-O (Ti2p) | Ti^4+^-O (Ti2p) | Ti-OH (O1s) |
| --- | --- | --- | --- | --- |
|  |  | 457.3 eV | 459.1 eV | 531.1 eV |
| Peak  percentage (%) | Anatase | 0.7 | 11.3 | 14.1 |
|  | A_d_ | 18.4 | 6.9 | 35.6 |

**Table S2.** The electronegativity of Ti, Sn, and C in Pauling scale.

| Elements | Ti | Sn | C |
| --- | --- | --- | --- |
| Electronegativity (Pauling scale) | 1.54 | 1.96 | 2.55 |

**Table S3.** The FT-IR peak assignments of SnO_2_[O]rTiO_2_-PGN.

| Wavenumber  [cm^-1^] | Assignments | Ref. |
| --- | --- | --- |
| 560 | SnO_2_/TiO_2_ | [33, 34] |
| 600 | SnO_2_/TiO_2_ | [33, 34] |
| 662 | Ti-O-Ti | [20] |
| 795 | Ti-O-C | [28, 35] |
| 1030 | Ti-O-Sn | [34] |
| 1438 | C=C, Aromatic | [36] |
| 1560 | C=C, Aromatic | [36] |
| 1635 | C=C, Alkene | [36] |
| 1730 | C=O | [35] |

**Table S4.** ICP-OES elemental quantitative analysis result of SnO_2_[O]rTiO_2_-PGN.

|  | Metal elements wt% | | Calculated composition | | |
| --- | --- | --- | --- | --- | --- |
| Species | Ti | Sn | TiO_2_ | SnO_2_ | PGN |
| Wt % | 20.2 | 16.3 | 33.7 | 20.7 | 45.6 |

**Table S5.** Summary of A_d_ rTiO_2_ and SnO_2_ mass ratio and the corresponding XRD characteristic ratio.

| **Numb.** | **Sample name** | **rTiO_2_/SnO_2_**  **(mass)** | **I_a_/I_b_** |
| --- | --- | --- | --- |
| 1 | SnO_2_[O]rTiO_2_-PGN | 6:3 | 3.6 |
| 15 | SnO_2_[O]rTiO_2_-PGN(II) | 6:6 | 4.5 |
| 16 | SnO_2_[O]rTiO_2_-PGN(III) | 6:9 | 4.7 |
| 17 | SnO_2_[O]rTiO_2_-PGN(IV) | 6:10 | 4.8 |
| 8 | SnO_2_-PGN | 6: ∞ | --- |

**Table S6.** Summary of achieved capacities of SnO_2_, TiO_2,_ and their composites in literature.

| **Numb.** | **Materials** | **Reversible capacity**  **(mAh g^-1^)** | **Current density** | **Reference** |
| --- | --- | --- | --- | --- |
| 1 | Ana. TiO_2_ NS | 175 | 1 C | Chen, J. S., et al., J Am Chem Soc (2010) 132, 6124^1^ |
| 2 | Mesoporous TiO_2_ | 210 | 1 C | Energy Environ. Sci. 2010, 3, 939–948^2^ |
| 3 | Titanate NS | 190 | 2 C | Adv. Mater., 2011, 23, 8, 998–1002^3^ |
| 4 | Black TiO_2_ | 200 | 0.2 C | Energy Environ. Sci., 2013, 6, 2609^4^ |
| 5 | 2D-TiO_2_ | 192 | 0.1 mA/g | Angew. Chem. Int. Ed. 2019, 58, 8740 –8745^5^ |
| 6 | TiO_2_/CNT | 300 | 0.1 mA/g | Chem. Mater. 2010, 22, 1908–1914^6^ |
| 7 | TiO_2_/graphene | 230 | 0.1 C | ACS Nano 2012, 6, 11035–11043^7^ |
| 8 | TiO_2_/C | 280 | 0.1 C | ACS Appl. Mater. Interface 2013, 5, 6478–6483^8^ |
| 9 | Sn-TiO_2_ | 300 | 0.1 C | J. Mater. Chem. A, 2013, 1, 13222^9^ |
| 10 | TiO_2_-SnO_2_ NTs | 345 | 0.1 mA/g | J. Mater. Chem. A, 2016, 2, 5542–5552^10^ |
| **11** | **SnO_2_[O]rTiO_2_-PGN** | **600** | **0.1 mA/g** | **This work** |

1 Chen, J. S. *et al.* Constructing Hierarchical Spheres from Large Ultrathin Anatase TiO2 Nanosheets with Nearly 100% Exposed (001) Facets for Fast Reversible Lithium Storage. *Journal of the American Chemical Society* **132**, 6124-6130, doi:10.1021/ja100102y (2010).

2 Saravanan, K., Ananthanarayanan, K. & Balaya, P. Mesoporous TiO2 with high packing density for superior lithium storage. *Energy & Environmental Science* **3**, 939-948, doi:10.1039/C003630G (2010).

3 Liu, J., Chen, J. S., Wei, X., Lou, X. W. & Liu, X.-W. Sandwich-Like, Stacked Ultrathin Titanate Nanosheets for Ultrafast Lithium Storage. *Advanced materials* **23**, 998-1002, doi:https://doi.org/10.1002/adma.201003759 (2011).

4 Myung, S.-T. *et al.* Black anatase titania enabling ultra high cycling rates for rechargeable lithium batteries. *Energy & Environmental Science* **6**, 2609, doi:10.1039/c3ee41960f (2013).

5 Yang, J. *et al.* Size-Independent Fast Ion Intercalation in Two-Dimensional Titania Nanosheets for Alkali-Metal-Ion Batteries. *Angew Chem Int Ed Engl* **58**, 8740-8745, doi:10.1002/anie.201902478 (2019).

6 Cao, F.-F. *et al.* Symbiotic Coaxial Nanocables: Facile Synthesis and an Efficient and Elegant Morphological Solution to the Lithium Storage Problem. *Chemistry of Materials* **22**, 1908-1914, doi:10.1021/cm9036742 (2010).

7 Xin, X., Zhou, X., Wu, J., Yao, X. & Liu, Z. Scalable Synthesis of TiO2/Graphene Nanostructured Composite with High-Rate Performance for Lithium Ion Batteries. *ACS Nano* **6**, 11035-11043, doi:10.1021/nn304725m (2012).

8 Wang, W. *et al.* Porous TiO2/C Nanocomposite Shells As a High-Performance Anode Material for Lithium-Ion Batteries. *ACS Applied Materials & Interfaces* **5**, 6478-6483, doi:10.1021/am402350n (2013).

9 Wang, Y., Xu, M., Peng, Z. & Zheng, G. Direct growth of mesoporous Sn-doped TiO2 thin films on conducting substrates for lithium-ion battery anodes. *Journal of Materials Chemistry A* **1**, 13222, doi:10.1039/c3ta13198j (2013).

10 Madian, M. *et al.* Anodically fabricated TiO2–SnO2 nanotubes and their application in lithium ion batteries. *Journal of Materials Chemistry A* **4**, 5542-5552, doi:10.1039/c6ta00182c (2016).
